# Launch sequencing of pharmaceuticals with multiple therapeutic indications: evidence from seven countries

**DOI:** 10.1186/s12913-023-09095-2

**Published:** 2023-02-13

**Authors:** Mackenzie Mills, Daniel Michaeli, Aurelio Miracolo, Panos Kanavos

**Affiliations:** 1 Department of Health Policy and LSE Health - Medical Technology Research Group (MTRG), London School of Economics, Houghton St, London, WC2A 2AE UK; 2 Fifth Department of Medicine, University Hospital Mannheim, Heidelberg University, Mannheim, Germany; 3 Department of Personalized Oncology, University Hospital Mannheim, Heidelberg University, Mannheim, Germany

**Keywords:** Oncology, Clinical Development, Regulatory Approval, HTA, Indication-based pricing

## Abstract

**Background:**

New medicines are increasingly being identified as efficacious across multiple indications. The impact of current pricing and reimbursement policies on launch decisions across these indications remains unclear.

**Objective:**

This paper, first, maps marketing authorisation and HTA coverage recommendation sequences of multi-indication medicines across Germany, France, England, Scotland, Canada, Australia, and the USA, and, second, evaluates the clinical characteristics, clinical development time and coverage recommendation time of multi-indication medicines, drawing comparisons between the first and subsequent indications of an approved molecule.

**Methods:**

Medicine approvals by the Food and Drug Administration between 2009–2019 were screened to identify multi-indication products with approved oncology indications. Data on clinical trial characteristics, clinical performance and HTA outcomes were extracted from publicly available regulatory approval and HTA reports.

**Results:**

Relative to subsequent indications, first indications were more likely to receive conditional marketing authorisation, have an orphan designation, have a single arm phase II pivotal trial and lower MCBS score. Subsequent indications had faster HTA coverage recommendation times in England and Canada. While the majority of first indications received HTA coverage recommendations across all settings, the proportion of subsequent indications with HTA coverage recommendations was lower and uptake varied considerably across settings.

**Conclusions:**

Discordance in the value of first versus subsequent indications can pose major challenges in systems that define price based on the initial indication. Current pricing and reimbursement systems generate significant fragmentation in the approval and availability of multi-indication products across settings.

**Supplementary Information:**

The online version contains supplementary material available at 10.1186/s12913-023-09095-2.

## Background

In 2018, over two-thirds of cancer medicines were approved for use in multiple indications [1]. Developing an established medicine for use in a new indication carries a number of advantages over de novo development, including reduced R&D costs and regulatory advantages in terms of secondary patents and extension of marketing exclusivity [2, 3]. Oncology medicines in particular may be good candidates for follow-on innovation, as similar underlying causes may be present across cancer types. Multi-indication products generate interesting challenges for health insurance systems, which typically assign prices at product level, rather than indication level. Given differences in disease stage, disease pathology, and available therapeutic alternatives, the added value a product provides can vary significantly across its respective therapeutic indications [4]. There has been considerable debate about the best method to finance multi-indication medicines [5–8]. Approaches include a single price per molecule, a weighted pricing model, differential discounting and indication-based pricing [5]. While economists argue that providing separate prices for each use of a molecule is the optimal approach for maximising welfare, most countries opt for indirect methods such as weighted pricing or differential discounting due to regulatory barriers and administrative burden [7, 8].

Indication-based pricing, also known as indication-specific pricing or multi-indication pricing, is a form of price discrimination whereby each indication for a molecule is priced separately according to the incremental value it provides above the standard of care in that particular indication. Under a single-price-per-molecule system, the price is anchored at the first indication launched for a molecule and manufacturers may not launch indications with lower incremental value in order to avoid price erosion and a loss of producer surplus in the higher value indication. Under an indication-based pricing model, price differentiation across indications ensures that the price is linked to the incremental value each indication provides relative to the standard of care. In theory, this removes incentives to withhold the launch in subsequent indications, which improve health, but not necessarily to the same extent as the first indication, increases the number of patients that have access to the medicine in question and maximises social welfare. Economists have argued both in favour and against this type of model for pricing pharmaceuticals [5, 8]. While indication-based pricing represents a method for manufacturers to maximise their producer surplus and the overall value they receive from a medicine [8], it can also provide short-term benefits in terms of increased patient access to medicines, and long-term benefits in terms of incentivising research and development of a wider range of therapeutic indications (including lower value indications) [9].

Countries have taken different approaches to differentiating the (therapeutic) value of multiple indications for a single medicine. While no countries implement a pure form of indication-based pricing (e.g. different list prices for each indication of a molecule), a number of indirect indication-based pricing policies have been implemented [10]. Specifically, there are four broad mechanisms for implementation of indication-based pricing: a) blended or weighted pricing, b) differential discounting, c) different brand names for different indications, or d) outcomes-based reimbursement models (See Additional file 1: Appendix for a full overview of indication-based pricing mechanisms).

Contrary to single indication drugs, the impact of current pricing and reimbursement strategies on manufacturer launch decisions and patient access to multiple indications remains unclear. Current literature exploring issues surrounding multi-indication medicines and indication-based pricing are limited to discussions on economic theory [5–8], simulations or economic evaluations of individual multi-indication medicines [6, 11], or reviews of pricing and reimbursement policies [10, 12].

In light of the above, the aim of the paper is to analyse the extent to which current pricing and reimbursement policies in select developed countries lead to indication launch sequencing, the order in which pharmaceutical firms develop, launch and market the use of medicines in different therapeutic indications across jurisdictions. The specific objectives are twofold: first, to map the marketing authorisation and HTA coverage recommendation sequence of multi-indication oncology medicines with the view to understanding patterns in indication launch and whether these hold across different health care systems. Second, to compare and contrast the first indication launched for a medicine, with the subsequent indications in terms of clinical trial characteristics, regulatory approval timelines, coverage decisions and HTA coverage recommendation timelines and access to market in order to understand how manufacturers prioritise development indications. By focusing on oncology indications, the paper contributes to the literature on indication-based pricing in 3 ways: first, it introduces a conceptual framework for the analysis of multi-indication medicines with specific focus on market entry dynamics and clinical characteristics; second, it provides a comprehensive empirical analysis with rich descriptive evidence on the clinical characteristics of multi-indication oncology medicines; and, third, it adopts a comparative and international perspective by examining marketing authorisation and HTA coverage recommendation patterns and sequence across selected countries in order to identify whether launch strategies vary depending on differences in regulatory settings or display similarities, despite these differences.

## Methods

### Conceptual framework

The launch of a specific indication for a medicine can be considered at both a global and local level. In the first instance, manufacturers must make a decision about whether to invest in research and development for a specific use of a new molecule. Global launch is triggered by receipt of marketing authorisation in at least one setting (often the U.S. Food and Drug Administration (FDA) is targeted first) [13]. Following development of a product for an indication and global launch (or at times in parallel to global launch), decisions are made about launch of the same product-indication in other markets. Local launch involves submission for MA and, depending on the context, may or may not require an HTA coverage recommendation.

Sequencing the launch of different indications is a function of the expected value of the indication and extent to which it contributes to return on investment and profit maximisation. Manufacturers may sequence the launch of different indications in two ways, which can be inter-connected: pre-development sequencing and post-development sequencing.

A Pre-development sequencing (pre-pivotal trial)

Pre-development sequencing relates to the decision on whether or not to pursue global launch for a particular indication and occurs prior to full development or submission to a regulatory authority. Under pre-development sequencing, manufacturers may prioritise initiation of a pivotal trial for indications with high perceived value for a specific molecule and may elect not to develop or delay the development of indications with a low perceived value. Early clinical data (or evidence of therapeutic advantage), price benchmarking, unmet need, and/or market size are factors which may contribute to the perceived value of an indication and influence decisions to develop and/or register a new medicine. Manufacturers may face a trade-off between price and market size and may opt to develop first in a niche or orphan designation, where budget constraints may be smaller, in order to establish a target benchmark price before expanding into indications with larger patient populations. The impact of competitors developing medicines in similar indications can also influence sequencing strategies.

B Post-development sequencing

Post-development sequencing relates to decisions to launch indications in local markets following development and global launch. A combination of clinical, economic and ethical considerations contribute to post-development decision making. Local launch decisions apply to both first and subsequent indications for a molecule, however price benchmarking and coverage of an initial indication within a specific setting may influence decisions to launch subsequent indications. Under a system where current pricing and reimbursement policies do not adequately capture the incremental value of individual indications, manufacturers may choose not launch of an indications. Typically, this could occur in cases where there are comparable alternative treatments available to patients and if the introduction of a subsequent indication would lead to substantial price erosion based on the presence of previous indications, lower perceived therapeutic advantage or higher uncertainty over therapeutic advantage. Manufacturers may adopt different strategies across countries based on variations in the perceived value of an indication across settings (e.g. due to differences in the HTA approaches or differences in unmet need). Decisions not to launch an indication in a particular jurisdiction can occur through one of three mechanisms: first, a manufacturer may elect not to submit for regulatory approval; second, a manufacturer may receive regulatory approval for an indication but elect not to submit for HTA review; and, third, a manufacturer may receive regulatory approval for an indication and submit for HTA review. If there is failure to reach agreement with a payor on an acceptable or cost-effective price (depending on the setting), the manufacturer may choose not to launch an indication.

Additional consideration is given to the nature of the multi-indication medicines being developed. Multi-indication medicines can be broadly grouped into three categories depending on the extent to which the various indications are similar. At the broadest level, a molecule can have multiple indications that span distinct therapeutic areas (e.g. oncology vs ophthalmology). Second, a molecule can have multiple indications across different diseases within a specific therapeutic area (e.g. melanoma vs lung cancer). Third, a molecule can have multiple indications that span different lines of therapy for a particular disease (e.g. 1^st^ line vs 2^nd^ line metastatic castrate resistant prostate cancer). The association between unmet need and market size may vary across type of multi-indication medicines. For molecules with multiple indications across different lines of therapy within a specific disease, unmet need tends to be highest in late stage relapsed/refractory patient populations that have exhausted other treatment alternatives. Fundamentally, the patient population in late stage disease will likely be smaller and clinical trials may be shorter for later-line therapies, with possible reduced life expectancy. However, the same association may not be present when developing indications across multiple types of cancer or across different therapeutic areas.

### Sample selection and data sources

FDA marketing authorisations were screened between January 1^st^, 2009 and January 1^st^, 2019 to identify a recent sample of multiple indication medicines that have launched globally. Medicines with a first approval after January 1^st^, 2009 and a second indication approved prior to January 1^st^, 2019, were identified. The study cut-off date was selected to provide sufficient follow-up time to track indication approvals after the initial approval. The scope of the study was restricted to multi-indication medicines used in oncology, a therapeutic area that is a) of high interest to decision-makers given burden of disease, high treatment costs and challenges in evidence development [14] and b) increasingly subject to follow-on indication [1]. The study scope is also restricted to multi-indication monotherapy treatments to limit the impact of combination therapies. Inclusion criteria were: 1) a minimum of one approved indication for the treatment of oncology during the study period (regardless of whether this is a first approval or subsequent); and 2) a minimum of two monotherapy indications approved during the study period. A flow chart detailing sample selection is included in Additional file 1: Appendix B.

The countries in scope included England, Scotland, France, Germany, Canada, Australia, and the USA. Country selection was based on public availability of marketing authorisation reports, public availability of health technology assessment (HTA) reports, and language (English, French, and German). Regulatory agency websites were screened to identify marketing authorisation reports for all indications approved for the included multi-indication medicines. This included the U.S. FDA [15], the European Medicines Agency (EMA) [16], Health Canada [17], and the Australian Therapeutic Goods Administration (TGA) [18]. Characteristics of pivotal clinical trials were screened via clinicaltrials.gov [19]. The European Society of Medical Oncology website was screened to identify corresponding evidence on the magnitude of clinical benefit scale (MCBS). Indications without an MCBS score were graded in accordance with the validated MCBS scorecard methodology based on clinical trial performance [20]. Finally, HTA agency websites were screened to identify HTA recommendations issued for all indications for the selected multi-indication medicines. This included the National Institute of Health and Care Excellence (NICE—England) [21], the Scottish Medicines Consortium (SMC – Scotland) [22], the Federal Joint Committee (G-BA—Germany) [23], the Haute Authorité de Santé, (HAS—France) [24], the Canadian Agency for Drugs and Technologies in Health (CADTH) [25], and the Pharmaceutical Benefits Advisory Committee (PBAC—Australia) [26]. Regulatory approvals and HTA approvals for included indications were tracked for an additional two years beyond the cut-off date for first approval (01/01/2019). The data collection cut-off date for the sample is 01/01/2021.

### Data extraction

For all included indications, and based on country-specific (regulatory and/or HTA) information, data extraction included general information (molecule name, brand name and therapeutic indication), regulatory variables (MA date, MA type, and orphan designation), clinical variables (study design of pivotal trial, pivotal trial size, pivotal trial initiation date, type of primary endpoint, primary endpoint outcome, and MCBS Score), and HTA variables (HTA outcome and whether a molecule has been approved for listing (L), listing with criteria or restrictions (LWC) or it has been rejected (do not list – DNL), HTA submission date (where available), and HTA recommendation date) (See Table 1).

**Table 1:** List of variables extracted

General Information	
** Variable**	Description
** Molecule name**	International Non-proprietary Name (INN) of medicine
** Brand name**	Company branded name of marketed medicine
** Therapeutic indication**	Approved therapeutic label of marketed medicine, designating the intended and authorised use of a medicine in a specific patient population. For the included molecules, all approved therapeutic indications recorded from each regulatory agency (FDA, EMA, Health Canada, TGA)
Regulatory Variables	
** Variable**	Description
** Marketing authorisation date**	The approval date for marketing authorisation of a specific medicine—indication pair (dd/mm/yyyy). Recorded for each regulatory agency across all included medicine—indication pairs
** Marketing authorisation type**	The type of marketing authorisation granted for a specific medicine—indication pair. Categorised as standard approval, priority review, or conditional authorisation. Standard approval includes FDA standard approval, EMA standard approval, TGA standard approval and Health Canada notice of compliance (NOC). Priority review includes FDA priority review, EMA accelerated assessment, TGA priority review, and Health Canada priority review. Conditional authorisation includes FDA accelerated approval, EMA conditional marketing authorisation, TGA provisional approval, and Health Canada, notice of compliance with conditions (NOC/C)
** Orphan designation**	Medicine – therapeutic indication received an orphan designation by relevant regulatory agency (0 = no, 1 = yes). Orphan designations indicate the therapeutic indication applies to a rare or orphan disease patient population. Orphan designation criteria vary across settings. The EMA and TGA orphan designations requires a prevalence of less than 5 in 10,000. The FDA orphan designation requires that the condition affects less than 200,000 in the USA. Health Canada does not have an orphan designation
Clinical Variables	
** Variable**	Description
** Study design of pivotal trial**	The study design of the pivotal trial used to support conditional regulatory approval. Study designs are classified according to study phase (phase I, phase II, phase III, phase IV, or N/A for non-interventional studies), study blinding (open label or double blind), randomisation (randomised or non-randomised/single arm), and comparators (placebo controlled, actively controlled or uncontrolled)
** Pivotal trial size**	The number of patients enrolled in the pivotal trial
** Pivotal trial initiation date**	The initiation date of the pivotal trial (per clinicaltrials.gov)
** Type of primary endpoint**	The type of primary endpoint used within the pivotal trial (0 = surrogate endpoint, 1 = clinical endpoint). Surrogate endpoints provide an indication or prediction of clinical benefit and provide early signals of a medicines efficacy. The following endpoints have been classified as surrogate within this study: progression free survival (PFS), overall response rate (ORR), subependymal giant cell astrocytoma (SEGA) volume, angiomyolipoma response rate, best observed response (BOR), Primary response (PR), Spleen volume reduction, remission free survival (RFS), complete response rate (CR), duration of response (DOR), major cytogenic response (MCyR), major molecular response (MMR) best-corrected visual acuity (BCVA), forced vital capcity (FVC). Clinical endpoints are hard clinical outcomes that provide an objective measure of clinical benefit. The following endpoints have been classified as clinical within this study: overall survival (OS), maintenance of vision, and seizure frequency
** Primary endpoint outcome**	The performance of the primary endpoint defined based on the trial protocol. Includes performance of active arm, performance of control arm, hazard ratio, confidence intervals, and significance (p value). For oncology indications, primary endpoints are predominantly either median progression-free survival (months) or median overall survival (months)
** MCBS Score**	The magnitude of clinical benefit scale (per www.esmo.org/guidelines/esmo-mcbs). The MCBS scale is 5 category ranking scale outlining the strength of evidence from 1 (low benefit) to 5 (high benefit). A ranking of 4 or 5 indicates substantial magnitude of benefit. The scale is based predominantly based on the performance of the primary endpoint, and is adjusted for quality of life improvements or changes in toxicity
HTA variables	
** Variable**	Description
** HTA Outcome**	HTA outcomes are classified as List (L), List with conditions (LWC), Do not list (DNL), or No HTA submission. In Germany, the G-BA added benefit ratings determine pricing, rather than the listing of a drug. We classify “lesser benefit” or “no proof of added benefit” ratings as DNL, “Proof of major or significant added benefit” as L, and all other ratings as LWC. Note that medicines with lesser or no proof of added benefit may still be reimbursed in Germany based on reference pricing. In France, the medical service rendered (SMR) rating determines the rate of reimbursement, while the improvement in medical service rendered (ASMR) determines pricing. We classify medicines with an SMR of insufficient as DNL, medicines with an SMR of Important and an ASMR of Major or Important as L, and all other ratings as LWC
** HTA recommendation date**	The HTA coverage recommendation date (dd/mm/yyyy)
** HTA submission date**	The date in which manufacturers filed their submission for health technology assessment. Only available for NICE, SMC and CADTH

Source: The authors

*Abbreviations: CADTH* Canadian Agency for Drugs and Technologies in Health, *EMA* European Medicines Agency, *FDA* Food and Drug Administration (USA), *GBA* Federal Joint Committee (Germany), *HAS* Haute Autorité de Santé (France), *HC* Health Canada, *HTA* health technology assessment, *NICE* National Institute of Health and Care Excellence (England and Wales), *PBAC* Pharmaceutical Benefits Advisory Committee (Australia), *SMC* Scottish Medicines Consortium (Scotland), *TGA* Therapeutic Goods Administration (Australia)

### Analysis

Data was extracted into a dataset in Microsoft Excel and imported into STATA SE Version 15.1. for analysis. The first indication for a molecule to receive FDA approval was classified as the “first indication” and all subsequent indications were classified as “subsequent indications”.

The following research endpoints were studied: first, *the alignment between global launch sequence, national regulatory approval and HTA recommendation sequence* was examined through a mapping of global launch, regulatory approval and HTA recommendation in each of study countries. For each molecule, codes were assigned to each indication launched based on launch sequence across FDA, EMA, Health Canada, and TGA. The global launch date (date of first approval in one of FDA, EMA, Health Canada or TGA), total number of distinct indications identified, the proportion of indications with regulatory approval in each jurisdiction and the proportion of indications with positive HTA coverage recommendations are tabulated to identify differences in regulatory and HTA approval across settings. Separately, the global launch sequence and HTA approval sequence are compared and tabulated in order to identify instances of post-development sequencing.

Second, *differences in regulatory approval and clinical characteristics of first vs second indications* were explored through descriptive statistics with the aim of understanding how manufacturers are prioritising development of indications. The regulatory approval pathway and regulatory designations provide an indication of the extent to which disease severity, unmet need, and market size are prioritised. Clinical characteristics are considered in terms of quality of clinical evidence (pivotal trial design, trial size, and type of primary endpoint) and the MCBS score of a medicine, which provides an aggregate measure of the strength and quality of evidence. Categorical variables (MA type, orphan designation, trial design, type of endpoint, and MCBS score) were analysed using Pearson Chi-squared tests. Mean trial size was analysed using two sample t-tests.

Third, *differences in HTA outcome of first vs subsequent indications* are also explored through descriptive statistics (Pearson Chi-Squared tests) to identify whether subsequent indications are less likely to receive HTA coverage recommendations. Additionally, the association between HTA outcome and MCBS score is calculated for each HTA agency (Pearson Chi-Squared test).

Fourth, *clinical development time and HTA coverage recommendation time* was evaluated through survival analysis through a comparison first and subsequent indications. Clinical development time was defined as T_1_-T_0_, where T_0_ represents pivotal clinical trial initiation date, and T_1_ represents MA approval date. T_1_ is defined for each country based on the relevant regulatory agency, such that clinical development time (T_1_-T_0_) for a specific medicine-indication pair may vary across Europe, Canada, Australia, and the FDA. HTA coverage recommendation timelines were defined as T_2_-T_1_, where T_2_ represents HTA coverage recommendation date. Kaplan Meier plots were produced for both clinical development time and HTA coverage recommendation time. Log-rank tests were used to identify differences in survival plots of first indications and subsequent indications. Subgroup analysis was performed at country level and according to type of multi-indication medicine. Additional analysis was performed to evaluate time from marketing authorisation to HTA submission for CADTH, G-BA and NICE, where data on HTA submissions is available. Mean time from marketing authorisation to HTA submission for first vs subsequent indications was calculated using two sample t-tests.

## Results

### Sample overview

Out of 90 multi-indication medicines identified in the study period, 31 medicines met the inclusion criteria for the study (See Additional file 1: Appendix B). Of these 31 medicines a total of 118 distinct therapeutic indications were identified. Four medicines had multiple indications approved across therapeutic areas ibrutinib, nintedanib, aflibercept, and everolimus) corresponding to 18% of total indications (*n* = 22). Sixteen medicines had multiple indications across different types of cancer (cabozantinib, pazopanib, tisagenlecleucel, regorafenib, remucirumab, avelumab, atezolizumab, eribulin, ruxolitinib, nivolumab, pembrolizumab, brentuximab vedotin, ipilimumab, romidepsin, vemurafenib, and lenvatanib), corresponding to 58% indications (*n* = 68). Eleven medicines had multiple indications across different lines of therapy within the same disease (abiraterone acetate, afatinib, blinatumomab, enzalutamide, rucaparib, osimertinib, crizotinib, bosutinib, alectinib, and ceritinib, ofatumumab), corresponding to 24% of total indications (*n* = 28). Out of the 118 indications identified, 32 were classified as “first indications” and 86 were classified as “subsequent indications” (brentuxiumab vedotin had two initial indications approved). A full list of indications included is provided in Additional file 1: Appendix C.

### Sequence alignment between global launch, national regulatory approval and HTA recommendation

The FDA approved the highest proportion of indications, with 115 approvals (97%) followed by the EMA with 96 approvals (81%), Health Canada with 94 (80%) and TGA with 93 (79%). In a limited number of cases, applications for marketing authorisations were withdrawn (5 indications for EMA, 1 indication for Health Canada, and 1 indication for TGA) or refused (1 indication for EMA. The first launch of each indication was predominantly in the FDA (106 indications had their first approval in the FDA vs 12 in the EMA, and 0 in Health Canada or the TGA).

HTA outcomes for multi-indication products were highly variable at both indication and molecule level. No multi-indication medicine had a positive HTA coverage recommendation for all globally launched indications. First indications had a high frequency of positive HTA recommendations across settings. Out of 32 first indications evaluated, positive recommendations were identified for 29 (91%) by Germany, 28 (88%) by HAS, 27 (84%) by NICE, 26 (81%) by SMC, 25 (78%) by PBAC and 23 (72%) by CADTH. Subsequent indications had a lower frequency of positive HTA recommendations across all settings. Out of 86 subsequent indications evaluated, positive HTA recommendations were identified for 60 (70%) by HAS, 58 (67%) by Germany, 48 (56%) by NICE, 50 (58%) by SMC, 50 (58%) by PBAC, and 51 (59%) by CADTH (See Table 2).

**Table 2:** Marketing authorisation and hta approvals of multi-indication oncology products in England, Scotland, France, Germany, Ontario, and Australia

		**Regulatory Approvals**					**HTA Approvals**					
**Molecule**	**First indication approval date**^**a**^	**Total number of distinct indications identified**^**b**^	**FDA approvals** **n (%)**	**EMA** **approvals** **n (%)**	**HC** **approvals** **n (%)**	**TGA approvals** **n (%)**	**NICE approvals** **n (%)**	**SMC approvals** **n (%)**	**HAS approvals**^**c**^ **n (%)**	**GBA approvals**^**d**^ **n (%)**	**CADTH approvals** **n (%)**	**PBAC approvals** **n (%)**
Ibrutinib	13/11/2013	**6**	6 (100%)	4 (67%)	6 (100%)	4 (67%)	3 (50%)	3 (50%)	4 (67%)	3 (50%)	3 (50%)	3 (50%)
Nintedanib	25/09/2014	**2**	1 (50%)	2 (100%)	1 (50%)	2 (100%)	2 (100%)	2 (100%)	1 (50%)	2 (100%)	1 (50%)	1 (50%)
Aflibercept	20/09/2011	**7**	5 (71%)	6 (86%)	6 (86%)	6 (86%)	5 (71%)	6 (86%)	6 (86%)	1 (14%)	4 (57%)	5 (71%)
Everolimus	30/03/2009	**7**	7 (100%)	7 (100%)	7 (100%)	6 (86%)	4 (57%)	3 (43%)	7 (100%)	1 (14%)	3 (43%)	5 (71%)
Cabozantinib	29/11/2012	**4**	4 (100%)	4 (100%)	3 (75%)	3 (75%)	3 (75%)	1 (25%)	3 (75%)	3 (75%)	2 (50%)	1 (25%)
Pazopanib	19/10/2009	**2**	2 (100%)	2 (100%)	2 (100%)	2 (100%)	1 (50%)	1 (50%)	1 (50%)	0 (0%)	1 (50%)	2 (100%)
Tisagenlecleucel	30/08/2017	**2**	2 (100%)	2 (100%)	2 (100%)	2 (100%)	2 (100%)	2 (100%)	2 (100%)	2 (100%)	0 (0%)	0 (0%)
Regorafenib	27/09/2012	**3**	3 (100%)	3 (100%)	3 (100%)	3 (100%)	2 (67%)	2 (67%)	3 (100%)	2 (67%)	2 (67%)	0 (0%)
Ramucirumab	21/04/2014	**3**	3 (100%)	3 (100%)	1 (33%)	1 (33%)	0 (0%)	0 (0%)	2 (67%)	1 (33%)	1 (33%)	1 (33%)
Avelumab	23/03/2017	**2**	2 (100%)	1 (50%)	2 (100%)	1 (50%)	1 (50%)	1 (50%)	1 (50%)	1 (50%)	1 (50%)	1 (50%)
Atezolizumab	18/05/2016	**3**	3 (100%)	3 (100%)	3 (100%)	3 (100%)	3 (100%)	1 (33%)	2 (67%)	2 (67%)	1 (33%)	2 (67%)
Eribulin	15/11/2010	**2**	2 (100%)	2 (100%)	2 (100%)	2 (100%)	1 (50%)	1 (50%)	2 (100%)	1 (50%)	1 (50%)	2 (100%)
Ruxolitinib	16/11/2011	**2**	2 (100%)	2 (100%)	2 (100%)	2 (100%)	1 (50%)	1 (50%)	2 (100%)	2 (100%)	2 (100%)	1 (50%)
Nivolumab	22/12/2014	**13**	13 (100%)	10 (77%)	10 (77%)	11 (85%)	9 (69%)	9 (69%)	9 (69%)	7 (54%)	8 (62%)	7 (54%)
Pembrolizumab	04/09/2014	**16**	16 (100%)	11 (69%)	10 (63%)	12 (75%)	8 (50%)	8 (50%)	8 (50%)	7 (44%)	8 (50%)	7 (44%)
Brentuximab vedotin	19/08/2011	**6**	6 (100%)	5 (83%)	6 (100%)	4 (67%)	3 (50%)	3 (50%)	4 (67%)	3 (50%)	6 (100%)	4 (67%)
Ipilimumab	25/03/2011	**5**	5 (100%)	3 (60%)	3 (60%)	3 (60%)	3 (60%)	3 (60%)	3 (60%)	2 (40%)	3 (60%)	3 (60%)
Romidepsin	01/05/2009	**2**	2 (100%)	0 (0%)	1 (50%)	1 (50%)	0 (0%)	0 (0%)	0 (0%)	0 (0%)	1 (50%)	0 (0%)
Vemurafenib	17/08/2011	**2**	2 (100%)	1 (50%)	1 (50%)	1 (50%)	1 (50%)	1 (50%)	1 (50%)	1 (50%)	0 (0%)	0 (0%)
Lenvatanib	13/02/2015	**3**	3 (100%)	3 (100%)	3 (100%)	3 (100%)	3 (100%)	3 (100%)	1 (33%)	2 (67%)	2 (67%)	2 (67%)
Abiraterone Acetate	28/04/2011	**3**	3 (100%)	3 (100%)	3 (100%)	3 (100%)	2 (67%)	3 (100%)	3 (100%)	3 (100%)	1 (33%)	1 (33%)
Afatinib	12/07/2013	**2**	2 (100%)	2 (100%)	2 (100%)	2 (100%)	1 (50%)	1 (50%)	1 (50%)	1 (50%)	1 (50%)	1 (50%)
Blinatumomab	03/12/2014	**3**	3 (100%)	3 (100%)	3 (100%)	3 (100%)	2 (67%)	3 (100%)	2 (67%)	3 (100%)	2 (67%)	2 (67%)
Enzalutamide	31/08/2012	**3**	3 (100%)	3 (100%)	3 (100%)	3 (100%)	2 (67%)	2 (67%)	3 (100%)	2 (67%)	3 (100%)	1 (33%)
Rucaparib	19/12/2016	**2**	2 (100%)	2 (100%)	0 (0%)	0 (0%)	1 (50%)	1 (50%)	1 (50%)	0 (0%)	0 (0%)	0 (0%)
Osimertinib	13/11/2015	**2**	2 (100%)	2 (100%)	2 (100%)	2 (100%)	1 (50%)	1 (50%)	2 (100%)	1 (50%)	2 (100%)	1 (50%)
Crizotinib	26/08/2011	**3**	3 (100%)	3 (100%)	2 (67%)	3 (100%)	3 (100%)	3 (100%)	3 (100%)	2 (67%)	2 (67%)	2 (67%)
Bosutinib	04/09/2012	**2**	2 (100%)	2 (100%)	2 (100%)	1 (50%)	1 (50%)	1 (50%)	2 (100%)	1 (50%)	1 (50%)	1 (50%)
Alectinib	11/12/2015	**2**	2 (100%)	2 (100%)	2 (100%)	2 (100%)	1 (50%)	1 (50%)	2 (100%)	2 (100%)	2 (100%)	1 (50%)
Ceritinib	29/04/2014	**2**	2 (100%)	2 (100%)	2 (100%)	2 (100%)	2 (100%)	1 (50%)	2 (100%)	0 (0%)	1 (50%)	1 (50%)
Ofatumumab	26/10/2009	**4**	4 (100%)	0 (0%)	0 (0%)	2 (50%)	0 (0%)	0 (0%)	0 (0%)	0 (0%)	0 (0%)	1 (25%)

*Abbreviations: CADTH* Canadian Agency for Drugs and Technologies in Health, *EMA* European Medicines Agency, *FDA* Food and Drug Administration (USA), *GBA* Federal Joint Committee (Germany), *HAS* Haute Autorité de Santé (France), *HC* Health Canada, *HTA* health technology assessment, *NICE* National Institute of Health and Care Excellence (England and Wales), *PBAC* Pharmaceutical Benefits Advisory Committee (Australia), *SMC* Scottish Medicines Consortium (Scotland), *TGA* Therapeutic Goods Administration (Australia)

^a^The date in which a molecule first received a marketing authorisation in one of the FDA, EMA, TGA or Health Canada. A detailed list of included indications is provided in Additional file 1: Appendix C

^b^The total number of distinct indications identified with approval in one or more of the FDA, EMA, TGA or Health Canada for a specific multi-indication molecule during the study period (01/01/2009 – 01/01/2019)

^c^In France, indications which receive an SMR rating of insufficient are categorized as having a negative HTA outcome (DNL)

^d^In Germany, indications which receive a rating of lesser benefit or no proof of added benefit are categorized as having a negative HTA outcome (DNL). In practice, these indications may still be reimbursed at a price determined based on reference pricing, and the HTA approval sequence does not necessarily reflect the order in which indications are launched within the country

Concordance between global launch sequence (defined based on first approval in one of FDA, EMA, Health Canada and TGA) and HTA coverage recommendation sequence was variable (See Table C2 in Additional file 1: Appendix C).

Medicines with multiple indications across distinct therapeutic areas ( typically received HTA coverage recommendations in a similar sequence to global launch sequence. Exceptions included ibrutinib, where the second indication launched globally (chronic lymophicytic leukemia) was approved by NICE, CADTH and PBAC prior to the first indication launched globally (mantle cell lymphoma) and everolimus, where the 5^th^ indication approved globally (advanced breast cancer) was the first to receive NICE approval and the 2^nd^ indication approved globally (subependymal giant cell astrocytoma) was the first to receive PBAC approval.

Concordance between global launch sequence and HTA coverage recommendation sequence for medicines with multiple indications across different oncologic diseases was mixed. HTA coverage recommendation of indications for pazopanib, tisagenlecleucel, regorafenib, ramucirumab, avelumab, eribulin, ruxolitinib, and ipilimumab typically followed global launch sequence, although a number of indications for all molecules were not approved. Cabozantinib, ibrutinib, nivolumab, pembrolizumab, brentuximab vedotin, and lenvatinib all had instances of global launch sequence not matching HTA recommendation sequence.

Medicines with multiple indications across different lines of therapy within a disease had a lower average number of indications. Concordance between HTA coverage recommendation sequence and global launch sequence was high, although a number of indications either were either not assessed by HTA agencies or received a negative recommendation.

### Differences in regulatory approval and clinical characteristics of first vs subsequent indications

First and subsequent indications were compared in terms of type of MA, orphan status, pivotal trial design, type of primary endpoint, trial size, MCBS score and HTA outcomes (See Table 3).

**Table 3:** Clinical evidence characteristics and hta outcomes of first vs subsequent indications

Category	Variable	First indication n (%)	Subsequent indication n (%)	*P* value
***REGULATORY APPROVAL***				
Type of MA granted (all agencies)	Standard	61 (51%)	207 (74%)	0.001
	Conditional	34 (29%)	39 (14%)	
	Priority review	24 (20%)	33 (12%)	
Type of MA granted (excluding EMA)	Standard	47 (53%)	146 (69%)	
	Conditional	24 (27%)	36 (16%)	0.032
	Priority review	18 (20%)	31 (15%)	
Orphan Designation^a^	Yes	55 (46%)	65 (23%)	< 0.0001
	No	64 (54%)	214 (77%)	
***CLINICAL EVIDENCE***				
Pivotal trial design	Phase II single arm	42 (35%)	56 (20%)	0.009
	Phase III placebo RCT	30 (25%)	76 (27%)	
	Phase III head-to-head	39 (33%)	129 (46%)	
	Other	8 (7%)	18 (6%)	
Type of primary endpoint	Clinical	28 (24%)	49 (18%)	
	Surrogate	81 (68%)	194 (69%)	0.221
	Co-primary	10 (8%)	36 (13%)	
Trial size	Number of enrolled patients Mean [ 95% CI]	486 [421 – 550]	555 [504 -605]	0.125
MCBS^b^	Score of 1	54 (48%)	88 (35%)	0.012
	Score of 2 or 3	22 (20%)	85 (34%)	
	Score of 4 or 5	35 (32%)	80 (31%)	
***HTA OUTCOMES***^c^				
G-BA	Proof of added benefit	25 (86%)	26 (45%)	0.004
	Lesser/no added benefit	4 (14%)	32 (57%)	
HAS	Reimbursed	27 (96%)	54 (90%)	0.299
	Not-reimbursed	1 (4%)	6 (10%)	
NICE	List/List with Criteria	26 (96%)	43 (90%)	0.304
	Do not list	1 (4%)	5 (10%)	
SMC	List/List with Criteria	23 (88%)	43 (86%)	0.763
	Do not list	3 (12%)	7 (14%)	
CADTH	List/List with Criteria	22 (96%)	41 (84%)	0.152
	Do not list	1 (4%)	8 (16%)	
PBAC	List/List with Criteria	23 (92%)	33 (66%)	0.015
	Do not list	2 (8%)	17 (34%)	

*p*-values calculated based on χ2 -test (for categorical variables) and two sample t-tests (for mean comparisons)

*Abbreviations: CADTH* Canadian Agency for Drugs and Technologies in Health, *GBA* Federal Joint Committee (Germany), *HAS* Haute Autorité de Santé (France), *HTA* health technology assessment, *MA* marketing authorisation, *NICE* National Institute of Health and Care Excellence (England and Wales), *PBAC* Pharmaceutical Benefits Advisory Committee (Australia), *PFS* progression-free survival, *SMC* Scottish Medicines Consortium (Scotland), *TGA* Therapeutic Goods Administration (Australia)

^a^Results presented are aggregated across all countries. The requirements for orphan designations vary across settings. For the FDA, the disease or condition must (A) affect less than 200,000 persons in the United States, or (B) affect more than 200,000 in the United States and for which there is no reasonable expectation that the cost of developing and making available in the United States a drug for such disease or condition will recovered from sales in the United States of such drug. For the EMA, the prevalence of condition in the EU must not be more than 5 in 10,000 or it must be unlikely that marketing of the medicine would generate sufficient returns to justify the investment needed for its development. For the TGA, one of the following criteria must apply: a) the condition affects fewer than 5 in 10,000 individuals in Australia when the application is made; b) if it were included in the Register, would not be likely to be supplied to more than 5 in 10,000 individuals in Australia during each year that it is included in the Register; or c) it is not likely to be financially viable for the sponsor to market the medicine in Australia. Health Canada does not have an orphan designation

^b^The magnitude of clinical benefit scale is a ranking of clinical benefit derived by the European Society for Medical Oncology, to grade the magnitude of benefit provided by a clinical trial. Ranking range from 1 (low) to 5 (high) clinical benefit. MCBS scores are grouped in terms of low benefit (1), moderate benefit (2 or 3) and substantial benefit (4 or 5) [20]

^c^Excludes indications that are not submitted for HTA approval. In Germany, indications which receive a rating of lesser benefit or no proof of added benefit are categorized as having a negative HTA outcome (DNL). In practice, these indications may still be reimbursed at a price determined based on reference pricing, and the HTA approval sequence does not necessarily reflect the order in which indications are launched within the country. In France, indications which receive an SMR rating of insufficient are categorized as having a negative HTA outcome (DNL)

Relative to subsequent indications, first indications were more likely to be approved based on a conditional marketing authorisation pathway (34 of 119 (29%) first indications vs 39 of 279 (14%) subsequent indications, *p* = 0.001). These results remain significant when excluding EMA from analysis (where conditional approval is only available for first-indications). First indications are also more likely to have an orphan designation (55 of 119 (46%) vs 65 of 279 (23%), *p* < 0.001) more likely to have a phase II single arm trial design (42 of 119 (35%) vs 56 of 279 (20%), *p* = 0.009), and are more likely to receive a low MCBS score (54 of 111 (48%) vs 88 of 253 (35%), *p* = 0.012). MCBS scores within individual multi-indication drugs were highly variable across indications (see Additional file 1: appendix table C1), with only 11 (33%) of medicines showing similar scoring across indications (everolimus, tisagenlecleucel, ramucirumab, avelumab, eribulin, rucolitinib, romidepsin, lenvatinib, blinatumomab, abiraterone, and bosutinib). No significant differences were identified between first and subsequent indications, for type of endpoint, trial size.

Subgroup analysis by type of multi-indication medicine was consistent with aggregate results with the following exceptions. Relative to subsequent indications, first indications for medicines with multiple indications across different therapeutic areas no longer show statistical significance for conditional approval (3 of 16 (18.75%) vs 6 of 60 (10%), *p* = 0.423), phase II pivotal trial design (*n* = 3 of 16 (18.75%) vs 10 of 60 (16.67), *p* = 0.505) or low MCBS score (3 of 8 (37.50%) vs 7 of 34 (21%), *p* = 0.418) and are more have a larger number of average patients in the pivotal trial (591 vs 371, *p* = 0.031). Relative to subsequent indications, first indications for medicines with multiple oncologic indications have a lower number of average patients in the pivotal trial (469 vs 588, *p* = 0.039) and no longer show significance for conditional approval (15 of 63 (23%) vs 29 of 163 (18%), *p* = 0.144 or low MCBS score (25 of 63 (40%) vs 62 of 163 (38%), *p* = 0.682). Relative to subsequent indications, first indications for medicines with multiple indications across different lines of therapy no longer show statistical significance for orphan designation (15 of 40 (38%) vs 18 of 56 (32%), *p* = 0.584).

### Differences in HTA outcome of first vs subsequent indications

With the exception of Australia and Germany, no significant differences were identified in HTA outcomes across settings, defined as the proportion of medicines evaluated by HTA agencies that received a positive HTA recommendation. In Australia, first indications were more likely to receive a positive recommendation: 23 of 25 (92%) of first indications evaluated vs 33 of 50 (66%) subsequent indications evaluated (*p* = 0.015). Within Germany, first indications were more likely to show evidence of added benefit than subsequent indications: 25 of 29 (86%) first indications evaluated vs 26 of 58 (45%) subsequent indications evaluated (*p* = 0.04).

Within Germany and France, HTA outcomes are significantly associated with MCBS score. In Germany 25 of 56 (45%) of indications with a low or moderate MCBS score (1, 2 or 3) received a rating of no added benefit vs 5 of 31 (16%) with an MCBS score of 4 or 5, *p* = 0.007. Within France 7 of 44 (14%) indication with a low or moderate MCBS score (1, 2 or 3) received an SMR of insufficient vs 0 of 28 (0%), *p* = 0.040. No significant differences in HTA outcome vs MCBS score were identified in NICE, SMC, CADTH or PBAC.

### Clinical development time and HTA coverage recommendation time

**Fig. 1:**
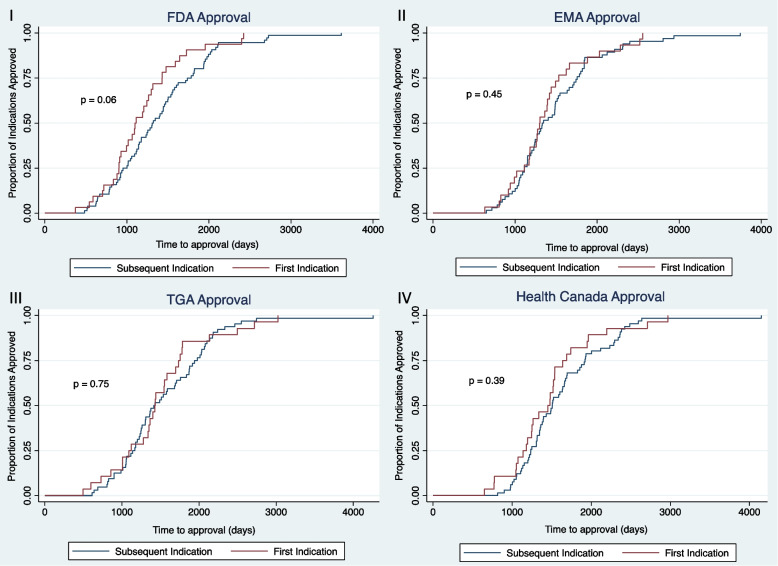
Kaplan Meier plots of clinical development time for multi-indication products, defined as time from pivotal trial initiation to regulatory approval. I – Clinical development time of first vs subsequent indications in the USA. II – Clinical development time of first vs subsequent indications in Europe. III – Clinical development time of first vs subsequent indications in Canada. IV – Clinical development time of first vs subsequent indications in Australia. Abbreviations: EMA – European Medicines Agency, FDA – Food and Drug Administration (USA), TGA – Therapeutic Goods Administration (Australia)

Survival analysis of first and subsequent indications in terms of clinical development time did not yield any statistically significant differences between the two groups (See Fig. 1). Little to no separation of survival curves was seen in the USA, Europe, Canada, and Australia. Median clinical development times were fastest in the USA (median time 1,098 days vs 1,310 days, *p* = 0.06), followed by the EMA (median time 1,299 days vs 1,331 days, *p* = 0.45), the TGA (median time 1,426 days vs 1,413 days, *p* = 0.75) and Health Canada (median time 1,451 days vs 1,507 days, *p* = 0.39) for first vs subsequent indications respectively.

**Fig. 2:**
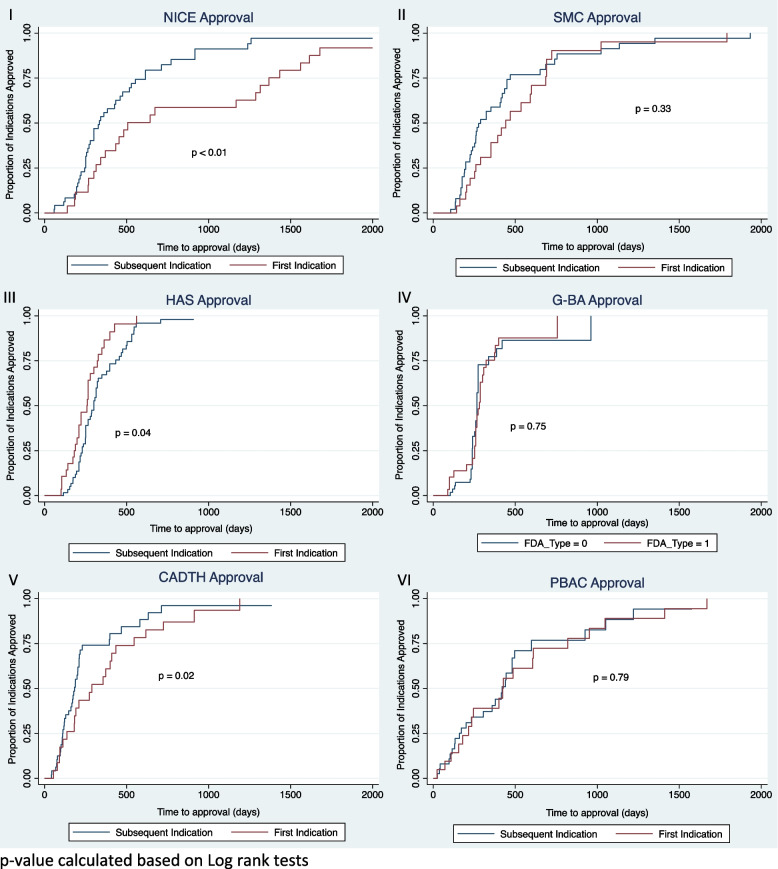
Kaplan Meier plots of HTA approval time for multi-indication products, defined as time from regulatory approval to HTA approval. I – HTA approval time of first vs subsequent indications in England. II – HTA approval time of first vs subsequent indications in Scotland. III – HTA approval time of first vs subsequent indications in France. IV – HTA approval time of first vs subsequent indications in Germany. V – HTA approval time of first vs subsequent indications in Canada. VI—HTA approval time of first vs subsequent indications in Australia. Abbreviations: CADTH—Canadian Agency for Drugs and Technologies in Health, G-BA – Federal Joint Committee, HAS – Haute Autorité de Santé (HAS), NICE – National Institute of Health and Care Excellence, PBAC – Pharmaceutical Benefits Advisory Committee, SMC – Scottish Medicines Consortium p-value calculated based on Log rank tests

HTA coverage recommendation timelines of first and subsequent indications varied across settings (See Fig. 2). In England and Canada, HTA coverage recommendation timelines were significantly longer for first indications than for subsequent indications. In England, median HTA coverage recommendation time was 506 days for first indications and 335 days for subsequent indications (*p* = 0.007). None of the indications studied were assessed under NICE’s fast track assessment procedure introduced in 2017. In Canada, median HTA coverage recommendation time was 289 days for first indications and 183 days for subsequent indications (*p* = 0.02). Within France, first-indications received approval marginally faster than subsequent indication (258 days vs 300 days, *p* = 0.04). No significant differences across first and subsequent indications were detected for HTA coverage recommendation timelines in Australia, Germany and Scotland.

HTA recommendation timelines were further evaluated in terms time from marketing authorisation to HTA submission across CADTH, G-BA, and NICE. Time from marketing authorisation to NICE submission was significantly longer for first indications vs subsequent indications (427 days vs 76 days, *p* = 0.01). No significant differences were detected across first vs subsequent indications for time from marketing authorisation to HTA submission in CADTH or G-BA, although time to submission was faster than NICE in both settings: 146 days for first indications vs 46 days for subsequent indications (*p* = 0.09) for CADTH; and 105 days for first indications vs 69 days for subsequent indications.

## Discussion

Decisions to prioritise the development of one indication for a medicine over another and decisions to launch a medicine in local settings following development are multifaceted. Manufacturers face a wide range of clinical, ethical and economic challenges when preparing a valuation for the new use of a medicine, which can vary significantly across both disease and country settings. From the evidence generated above it is clear that there is no uniform approach towards the development and marketing of multi-indication medicines. Nevertheless, a number of interesting observations can be identified in terms of how manufacturers are prioritising the development and launch of multi-indication medicines and in terms of how medicines become available in a given health care setting.

First, manufacturers show a tendency to prioritise development of niche indications, with high disease severity and unmet need for the first indication of multi-indication medicines. To a considerable degree this strategy seems to resonate with the objectives of health systems prioritising treatments that address significant unmet need and disease severity. Relative to subsequent indications, first indications were more likely to be based on conditional approval or priority review, indicative of a prioritisation of patient populations with high disease severity and unmet need for the first indication. Further, a higher proportion of first indications received an orphan designation. These results remain consistent when excluding EMA from analysis (where conditional approval is only granted to new drug submissions, rather than indication extensions).

Second, the evidence base of subsequent indications tends to be based on more robust study designs. Subsequent indications are more likely to be approved on the basis of phase III head-to-head trial designs, while first indications are more dependent on phase II, single arm trials. These findings are aligned with a higher proportion of conditional approvals and lower MCBS scores in first indications and a tendency to develop indications with high disease severity and unmet need in the first indication. In theory, development of indications that address unmet need and treat life threatening or chronically debilitating diseases can provide advantages to both patients, through access to a new treatment in the absence of therapeutic alternatives, and manufacturers, through lower requirements for market entry and reduced competition at the time of market entry. A further consideration relates to first-mover advantages, as manufacturers may prioritise development of indications which could result in being first to market, but based on less robust evidence of clinical evidence. However, no significant differences were detected in time from pivotal trial initiation to marketing authorisation across first and subsequent indications. In theory, development time would be shorter for subsequent indications if the safety and toxicity of a medicine has been established in the first indication [2]. However, this may not be reflected in the length of the pivotal trial, particularly if subsequent indications tend to be based on later phase clinical trials. Further research on earlier stages of clinical development could help to clarify this issue.

Third, while HTA coverage recommendation rates are similar across first and subsequent indications submitted for assessment, a number of indications do not launch in local settings. Mapping of marketing authorisation and HTA coverage recommendation sequence highlighted discordance between the total number of indications launched globally, the total number of indications with marketing authorisation individually within the EMA, TGA and Health Canada, and the total number of indications with HTA coverage recommendation. Results suggest that post-development sequencing typically manifests through non-launch of indications, frequently through absence of marketing authorisation. Only 81%, 80% and 79% of globally launched indications had authorisation in the EMA, TGA and Health Canada. Of the indications which did not launch, only a small number of withdrawals or refusals were identified, indicating that in most cases of non-approval manufacturers are electing not to submit for marketing approval. A number of authorised indications also failed to receive positive HTA coverage recommendations, however, with the exception of Australia and Germany, no significant differences were detected in HTA coverage recommendation rates across first and subsequent indications submitted for assessment.

HTA coverage recommendation sequence and HTA coverage recommendation rates should be interpreted with caution as variations in the role and scope of HTA are present across settings (See Table 4). Within England HTA recommendations by NICE are binding and positively recommended products must be made available within the NHS [27]. In Scotland, the SMC issues recommendations to NHS boards, who make final decisions on reimbursement [28]. In both settings, non-reimbursed products can still be purchased privately or be made available through private insurance schemes. Within Canada, reimbursement of medicines is primarily the responsibility of individual provinces, who rely on CADTH recommendations in an advisory capacity to inform pricing and reimbursement decisions [29]. Prior to launch, products are subject to an assessment by the Patented Medicines Pricing Review Board (PMPRB), who set a maximum allowable price, that applies to both the private and public market (where further discounts are negotiated) [30]. Similarly, PBAC in Australia serves an advisory role to the Ministry of Health for reimbursement in the Public Benefits Scheme [31]. Non-reimbursed products can be purchased privately following TGA approval. In Germany, new medicines are subject to the Act to Reorganize the Pharmaceuticals Market in the Statutory Health Insurance (AMNOG) procedure [32]. It is mandatory for newly marketed medicines to submit a benefit dossier with the Federal Joint Committee (G-BA) before commercialisation in Germany. Benefit assessment and subsequent price negotiations must take place within one year of authorisation. During this time medicines receive free pricing and are made available to patients [33, 34]. Finally, the HAS in France conducts HTA on all new drugs receiving marketing authorisation, and provides recommendations to the economic committee for healthcare products (CEPS), the national health insurance funds (UNCAM) and ministry of health [35].

**Table 4:** Role of HTA and requirements for launch of new indications across Germany, France, England, Scotland, Canada, and Australia

Country	HTA agency	Type of assessment	Role of HTA	Requirements for public reimbursement
Germany	Federal Joint Committee (G-BA)	Relative clinical benefit assessment	Informs pricing negotiations with the National Association of Statutory Health Insurance Funds	EMA authorisation
France	Transparency Committee—Haute Autorité de Santé (HAS)	Relative clinical benefit assessment	Informs pricing (ASMR) and reimbursement rate (SMR)	EMA authorisation and SMR rating above insufficient
England	National Institute of Health and Care Excellence (NICE)	Clinical and cost-effectiveness	Issues binding reimbursement recommendations. Indirectly influences pricing through cost-effectiveness thresholds	EMA authorisation and NICE approval^a^
Scotland	Scottish Medicines Consortium (SMC)	Clinical and cost-effectiveness	Informs pricing and reimbursement decisions by NHS boards. NHS boards not required to follow recommendation, but must wait for an SMC assessment to be issued	EMA authorisation and SMC assessment
Canada	Canadian Agency for Drugs and Technologies in Health (CADTH) and pan-Canadian Oncology Drug Review (pCODR)	Clinical and cost-effectiveness	Informs provincial pricing and reimbursement. Provinces are not required to follow recommendation and negotiate either jointly or individually with manufacturers	Health Canada authorisation and CADTH/PCODR assessment^b^
Australia	Pharmaceutical Benefits Advisory Committee (PBAC)	Clinical and cost-effectiveness	Informs pricing and reimbursement in Pharmaceutical Benefit Scheme (PBS). Minister of Health makes final decision following positive PBAC recommendation	TGA authorisation and PBAC approval

Source: The authors from [28–36]

*Abbreviations: ASMR* Amélioration du Service Médical Rendu (France), *EMA* European Medicines Agency, *HTA* Health Technology Assessment, *NHS* National Health Service, *SMR* Service Médical Rendu (France), *TGA* Therapeutic Goods Administration (Australia)

^a^NHS organisations are able to start using a new drug prior to NICE guidance but uptake is low and commissioning groups typically wait for NICE guidance to be issued. Since 2019, NICE evaluates all new drugs launched in the UK

^b^Provinces are able to fund drugs without CADTH/PCODR assessments but uptake is low and CADTH/PCODR recommendations typically inform negotiations. The province of Quebec does not rely on CADTH recommendations and has its own health technology assessment agency for informing pricing and reimbursement decisions

Finally, HTA coverage recommendation timelines tend to be faster for subsequent indications. Interestingly, subsequent indications had a tendency to have faster HTA coverage recommendation timelines, in England, France and Canada. This could partly be explained by higher quality pivotal clinical trial designs and increased proportion of standard approvals seen in the subsequent indication group. Another possibility is that first indications face higher barriers to entry. HTA agencies may receive efficiency gains from prior evaluations of a medicine in previous indications. Within England, differences in approval of first vs subsequent indications appears to be partly driven by delays in HTA submission of the first indication, perhaps indicating that manufacturers also receive efficiency gains in preparing HTA submissions for subsequent indications or alternatively reflecting increased challenges in preparing submissions with lower quality evidence and potentially higher uncertainty.

Our analysis is not without limitations. First, the present analysis is limited to indications that have received marketing approval, and thus no conclusions can be drawn about decisions not to develop indications pre-development; future research may explore this. Second, our analysis is limited to the USA, Europe, Canada and Australia. While these settings are frequently targeted first for global launch of medicines [13], we cannot exclude the possibility that medicines launch first in other jurisdictions. As such, it is possible that small differences exist between our classification of global launch sequence and true global launch sequence. Third, the results presented here predominantly reflect oncology medicines with multiple indications; further research is needed to establish whether our findings apply to multi-indication medicines in other therapeutic areas. Fourth, the impact of secondary patents and market exclusivity extensions was not explored in the analysis. The current patent regime enables drug innovators to pursue secondary patents for new uses of existing pharmaceuticals, while regulatory agencies may grant extensions in market exclusivity [37, 38]. These benefits may impact the timing of decisions to launch a product locally and could contribute to differences seen across settings in the timing and availability of indication extensions. Fifth, data was not collected on completion of confirmatory studies for conditional approvals. This may influence the strength of evidence at the time HTA evaluation or decisions to launch subsequent indications, an interesting topic that merits further research. Finally, reforms to HTA systems during the study period may influence results. For instance, the AMNOG process in Germany was not introduced until 2011, meaning no HTA reports were available prior to that [33]; further, NICE introduced reforms to their HTA timelines in 2016 as part of their change to the Cancer Drugs Fund (CDF), committing to processing all HTA submissions in 90 days after regulatory approval 28. This could contribute to the decrease in HTA coverage recommendation time and submission time seen for subsequent indications in England, but moreover, could influence launch decisions based on integration of the CDF into NICE recommendations 28.

## Conclusion

The development and marketing of multi-indication oncology medicines requires balancing a variety of factors that must be adjusted to the specific characteristics of a clinical setting. Manufacturers show a tendency to launch first in niche indications with high disease severity and unmet need, a strategy that seems to be compatible with what health systems demand, however, a number of examples are present of molecules which do not follow this trend. Of the 118 indications identified only 71% had marketing authorisation across each of the FDA, EMA, TGA and Health Canada, indicative of post-development sequencing. Substantial heterogeneity in HTA outcomes is present across settings although few significant differences were detected across first versus subsequent indications. Overall, discordance in the value of first vs subsequent indications can be a major challenge in systems that define price based on the initial indication, resulting in fragmented launch and availability of multi-indication products.

## Supplementary Information

**Additional file 1: Appendices A-C.**  

## Data Availability

All data sources used are publicly available. Regulatory agency websites were screened to identify marketing authorisation reports for all indications approved for the selected multi-indication medicines. This included the U.S. FDA, the European Medicines Agency (EMA), Health Canada, and the Australian Therapeutic Goods Administration (TGA). HTA agency websites were screened to identify HTA recommendations issued for all indications for the selected multi-indication medicines. This included the National Institute of Health and Care Excellence (NICE), the Scottish Medicines Consortium (SMC), the Federal Joint Committee (G-BA), the Haute Authorité de Santé, (HAS), the Canadian Agency for Drugs and Technologies in Health (CADTH), and the Pharmaceutical Benefits Advisory Committee (PBAC). Clinical trial characteristics were retrieved from clinicaltrials.gov. MCBS scores were retrieved from esmo.org.

## Abbreviations

CADTH: Canadian Agency for Drugs and Technologies in Health
DNL: Do not list
EMA: European Medicines Agency
FDA: Food and Drug Administration (U.S.)
GBA: Federal Joint Committee
HAS: Haute Authorité de Santé
HTA: Health Technology Assessment
L: Listed
LWC: Listed with Conditions
NICE: National Institute of Health and Care Excellence
NSCLC: Non-small cell lung cancer
PBAC: Pharmaceutical Benefits Advisory Committee
R&D: Research and Development
SMC: Scottish Medicines Consortium
TGA: Therapeutic Goods Administration (Australia)
U.S.: United States of America
