# Supplementary material for: Launch sequencing of pharmaceuticals with multiple therapeutic indications: evidence from seven countries
Source: BMC Health Serv Res. 2023 Feb 13;23:150. doi: 10.1186/s12913-023-09095-2 (PMC9923892; doi:10.1186/s12913-023-09095-2)
Supplement: Supplementary file 1 — Additional file 1: Appendices A-C. [file 12913_2023_9095_MOESM1_ESM.docx]

**Launch Sequencing of Pharmaceuticals with Multiple Therapeutic Indications: Evidence from Seven Countries**

*BMC Health Services Research*

**Mackenzie Mills^A^, MSc, Daniel Michaeli^A^, MSc, Aurelio Miracolo^A^, MSc, Panos Kanavos^A^, PhD**

A – Department of Health Policy and LSE Health, Medical Technology Research Group, London School of Economics and Political Science

**ADDRESS FOR CORRESPONDENCE**

Mackenzie Mills

Department of Health Policy

Medical Technology Research Group, LSE Health

Houghton Street London

WC2A 2AE

England

[M.J.Mills@lse.ac.uk](mailto:M.J.Mills@lse.ac.uk)

+44 (0) 7522725371

ORCID: 0000-0002-8756-3484

**APPENDIX A – COUNTRY SPECIFIC APPROACHES TO PRICING MULTI-INDICATION PRODUCTS.**

There are four broad mechanisms for implementation of indication-based pricing: a) blended or weighted pricing, b) differential discounting, c) different brand names for different indications, or d) outcomes-based reimbursement models

In some countries, such as Germany, France and Australia a weighted pricing system is used to indirectly achieve indication-based pricing. Under a weighted pricing system, the price of a medicine is re-negotiated after the launch of a new therapeutic indication [5]. Weighted pricing can apply to the list price of a molecule, or to the net price of a molecule in settings where confidential discounting takes place. The price is calculated based on the respective value of each indication and weighted according to the expected utilisation. In theory, this would generate the same level of sales as an indication-based pricing system. In practice, however, the weighted price may not reflect the true value or utilisation of respective indications (e.g. if a competitor launched in one indication and reduced utilisation). As a result, manufacturers may still be reluctant to launch lower value indications under a weighted pricing system unless a retrospective adjustment provision is in place to reflect actual patient volumes. This however, requires robust data capabilities and may be associated with high administrative burden [5].

In settings such as the UK, Switzerland and Italy different confidential discount rates can be applied off a single list price to individual indications for a molecule, particularly if the epidemiology or expected use varies substantially across indications. In these settings, regulatory and legal systems allow for the rate of reimbursement for a molecule to vary according to each indication’s value relative to the standard of care [11]. A payer’s willingness to provide differential discounts may be limited by issues in data capability and financial flow through distribution networks. Differential discounting requires tracking of a molecule’s use by indication and can be associated with substantial administrative burden. Wholesale distribution may lead to complexities in managing payments across indications [9].

Alternatively, regulatory and legal requirements may require that single price be used for each branded molecule [12]. Using different brand names for individual indications may provide flexibility to assign prices according to brand name. However, implementation of multiple brand names when indications are similar (e.g. for a molecule with multiple cancer indications) may be too confusing and burdensome for healthcare providers and patients [5]. Further, strict monitoring would be required to prevent off-label use of the lower-priced brand, a practice that is common in oncology where there is often high unmet need and patients may be more willing to use a medicine where definitive efficacy and safety have yet to be established [13].

Outcome-based reimbursement models have also been proposed as a potential solution to single pricing system. Outcome-based reimbursement models directly link payment to the real-world value that a medicine provides to patients. Effective outcomes-based reimbursement models could solve the disconnect between single payment models and the incremental value of multiple indications. In a recent study, authors evaluated the potential of outcomes-based payments to address issues in indication-based pricing of trastuzumab for breast cancer and for gastric cancer. Based on clinical trial efficacy, the expected value of trastuzumab in breast cancer and gastric cancer was $3.50 per mg and $0.93 per mg, respectively. However, based on data from an observational cohort, the expected value of trastuzumab in breast cancer and gastric cancer was $8.66 per mg and $0.20 per mg, respectively [6].

**APPENDIX B1 – SAMPLE SELECTION FLOWCHART**

**90 multi- indication medicines identified:**

First FDA indication approved after 01/01/2009 and a minimum of one indication approved prior to 01/01/2019

**42 multi-indication oncology medicines included**

Medicines with a minimum of one oncology indication approved in study period

**48 multi-indication medicines** with no oncology indications excluded

**31 multi-indication oncology medicines included**

Medicines with a minimum two monotherapy indications approved in study period

**11 multi-indication medicines** excluded based on combination therapies

**118** distinct indications identified

**402** matching MA reports identified

**479** matching HTA reports identified

**4 medicines** with multiple indications across distinct therapeutic areas

**16 medicines** with multiple indications across different types of cancer

**11 medicines** with multiple indications across different lines of therapy

**Figure B1 – Sample selection of medicines with multiple FDA approved therapeutic indications between January 1^st^, 2009 and January 1^st^, 2019.** Inclusion criteria included a) a minimum of one oncology/cancer indication and b) a minimum of two monotherapy indications. Following sample identification, matching marketing authorisation (MA) and health technology assessment (HTA) reports were identified across England, Scotland, France, Germany, Canada and Australia for included therapeutic indications. Marketing authorisation reports were identified from the U.S. Food and Drug Administration (FDA), the European Medicines Agency (EMA), Health Canada, and the Australian Therapeutic Goods Administration (TGA). HTA reports were identified from the National Institute of Health and Care Excellence (NICE), the Scottish Medicines Consortium (SMC), the Federal Joint Committee (G-BA), the Haute Authorité de Santé, (HAS), the Canadian Agency for Drugs and Technologies in Health (CADTH), and the Pharmaceutical Benefits Advisory Committee (PBAC).

**B2 – DATA SOURCES**

**Table B1 - Marketing Authorisation Report and Health Technology Assessment Report Data Sources**

| **Marketing Authorisation Agencies** | | |
| --- | --- | --- |
| **Country/Region** | **Agency** | **Website** |
| Europe | European Medicines Agency (EMA) | <https://www.ema.europa.eu/en> |
| USA | U.S. Food and Drug Administration (FDA) | <https://www.fda.gov/> |
| Canada | Health Canada | [https://www.canada.ca/en/ health-canada.html](https://www.canada.ca/en/%20health-canada.html) |
| Australia | Therapeutic Goods Administration (TGA) | <https://www.tga.gov.au/> |
| **HTA Agencies** | | |
| England | National Institute of Health and Care Excellence (NICE) | <https://www.nice.org.uk/> |
| Scotland | Scottish Medicines Consortium (SMC) | <https://www.scottishmedicines.org.uk/> |
| France | Haute Authorité de Santé, (HAS) | <https://www.has-sante.fr/> |
| Germany | Federal Joint Committee (G-BA) | <https://www.g-ba.de/english/> |
| Canada | Canadian Agency for Drugs and Technologies in Health (CADTH) | <https://www.cadth.ca/> |
| Australia | Pharmaceutical Benefits Advisory Committee (PBAC) | [https://www.pbs.gov.au/pbs/ind ustry/listing/participants/pbac](https://www.pbs.gov.au/pbs/ind%20ustry/listing/participants/pbac) |

**APPENDIX C – LIST OF INCLUDED MEDICINES AND INDICATIONS**

**TABLE C1 – LIST OF INCLUDED THERAPEUTIC INDICATIONS, FIRST APPROVAL DATE, PIVOTAL TRIAL DESIGN AND MCBS SCORES**

| Molecule name | Therapeutic Indication | First Marketing Authorisation Date | First Agency to Approve | Pivotal Trial Design | Primary Endpoint (s) | MCBS Score |
| --- | --- | --- | --- | --- | --- | --- |
| **MEDICINES WITH MULTIPLE INDICATIONS ACROSS DIFFERENT THERAPEUTIC AREAS** | | | | | | |
| Ibrutinib | For the treatment of patients with mantle cell lymphoma who have received at least one prior therapy | 13/11/2013 | FDA | Single Arm Phase II | ORR | 1 |
|  | For the treatment of Chronic lymphocytic leukemia who have received at least one prior therapy | 12/02/2014 | FDA | Phase III RCT - Active Comparator | PFS | 3 |
|  | Chronic lymphocytic leukemia with 17p deletion | 28/07/2014 | FDA | Phase III RCT - Active Comparator | PFS | 3 |
|  | Waldenström’s macroglobulinemia | 29/01/2015 | FDA | Single Arm Phase II | ORR | 1 |
|  | Marginal zone lymphoma who require systemic therapy and have  received at least one prior anti-CD20-based therapy | 18/01/2017 | FDA | Single Arm Phase II | ORR | 1 |
|  | Chronic graft versus host disease after failure of one or more lines of systemic therapy | 02/08/2017 | FDA | Single Arm Phase II | ORR | N/A |
| Nintedanib | In combination with docetaxel for the treatment of adult patients with locally advanced, metastatic or locally recurrent non-small cell lung cancer of adenocarcinoma tumour histology after first-line chemotherapy. | 25/09/2014 | EMA | Phase III RCT - Placebo Controlled | PFS | 2 |
|  | For the treatment of idiopathic pulmonary fibrosis. | 15/10/2014 | FDA | Phase III RCT - Active Comparator | FVC | N/A |
| Aflibercept | For the treatment of Neovascular (Wet) Age-Related Macular Degeneration | 20/09/2011 | EMA | Phase III RCT - Active Comparator | Maintenance of vision | N/A |
|  | In combination with 5-fluorouracil, leucovorin, irinotecan, is indicated for patients with metastatic colorectal cancer that is resistant to or has progressed following an oxaliplatin-containing regimen. | 03/08/2012 | FDA | Phase III RCT - Placebo Controlled | OS | 4 |
|  | For the treatment of Macular Edema following Central Retinal Vein Occlusion | 21/09/2012 | FDA | Phase III RCT - Active Comparator | BCVA change | N/A |
|  | For the treatment of visual impairment in adult patients with Diabetic Macular Edema (DME) | 26/06/2014 | EMA | Phase III RCT - Active Comparator | BCVA change | N/A |
|  | For the treatment of adult patients with visual impairment due to macular oedema secondary to branch retinal vein occlusion (BRVO) | 22/01/2015 | EMA | Phase III RCT - Active Comparator | BCVA change | N/A |
|  | For the treatment of patients with Diabetic Retinopathy (DR) in Patients with DME | 25/03/2015 | FDA | Phase III RCT - Active Comparator | BCVA change | N/A |
|  | For the treatment of visual impairment due to myopic choroidal neovascularisation (myopic CNV) | 24/09/2015 | EMA | Phase III RCT - Active Comparator | BCVA change | N/A |
| Everolimus | For the treatment of patients with advanced renal cell carcinoma after failure of treatment with sunitinib or sorafenib. | 30/03/2009 | FDA | Phase III RCT - Placebo Controlled | PFS | 3 |
|  | For the treatment of subependymal giant cell astrocytoma (SEGA) associated with tuberous sclerosis (TS) who require therapeutic intervention but are not candidates for curative surgical resection | 29/10/2010 | FDA | Single Arm Phase II | SEGA volume | N/A |
|  | For the treatment of progressive neuroendocrine tumors of pancreatic origin (PNET) that is unresectable, locally advanced or metastatic. | 05/05/2011 | FDA | Phase III RCT - Placebo Controlled | PFS | 3 |
|  | For the treatment of adults with renal angiomyolipoma and tuberous sclerosis complex (TSC), not requiring immediate surgery | 26/04/2012 | FDA | Phase III RCT - Placebo Controlled | angiomyolipoma response rate | N/A |
|  | For the treatment of hormone receptor-positive advanced breast cancer, in combination with an aromatase inhibitor, in postmenopausal women previously treated with endocrine therapy | 21/06/2012 | EMA | Phase III RCT - Placebo Controlled | PFS | 3 |
|  | For the treatment of non-functional neuroendocrine tumors (NET) of gastrointestinal (GI) or lung origin that are unresectable, locally advanced or metastatic. | 26/02/2016 | FDA | Phase III RCT - Placebo Controlled | PFS | 3 |
|  | For adjunctive treatment of patients aged 2 years and older with refractory seizures associated with tuberous sclerosis complex (TSC) | 15/12/2016 | FDA | Phase III RCT - Placebo Controlled | Seizure frequency | N/A |
| **MEDICINES WITH MULTIPLE THERAPEUTIC INDICATIONS ACROSS DIFFERENT TYPES OF CANCER** | | | | | | |
| Cabozantinib | For the treatment of adult patients with progressive, unresectable locally advanced or metastatic medullary thyroid carcinoma | 29/11/2012 | FDA | Phase III RCT - Placebo Controlled | PFS | 3 |
|  | For the treatment of advanced renal cell carcinoma (RCC) in patients who have received one prior therapy | 25/04/2016 | FDA | Phase III RCT - Active Comparator | PFS | 3 |
|  | For the treatment of advanced renal cell carcinoma the ‘treatment naïve adults with intermediate or poor risk per IMDC criteria | 19/12/2017 | FDA | Controlled Phase II | PFS | 2 |
|  | For the treatment of advanced hepatocellular carcinoma in adults following prior systemic therapy | 20/09/2018 | EMA | Phase III RCT - Placebo Controlled | OS | 4 |
| Pazopanib | For the treatment of patients with advanced renal cell carcinoma | 19/10/2009 | FDA | Phase III RCT - Active Comparator | PFS | 3 |
|  | For the treatment of patients with advanced soft tissue sarcoma who have received prior chemotherapy. | 26/04/2012 | FDA | Phase III RCT - Placebo Controlled | PFS | 3 |
| Tisagenlecleucel | For the treatment of patients up to 25 years of age with Bcell precursor acute lymphoblastic leukemia (ALL) that is refractory or in second or later  relapse. | 30/08/2017 | FDA | Single Arm Phase II | ORR | 1 |
|  | For the treatment of adult patients with relapsed or refractory (r/r) large B-cell lymphoma after two or more lines of systemic therapy including diffuse large B-cell lymphoma (DLBCL) not otherwise specified, high grade B-cell lymphoma and DLBCL arising from follicular lymphoma | 01/05/2018 | FDA | Single Arm Phase II | ORR | 1 |
| Regorafenib | For the treatment of patients with metastatic colorectal cancer (CRC) who have been previously treated with fluoropyrimidine-, oxaliplatin- and irinotecan-based chemotherapy, an anti-VEGF therapy, and, if KRAS wild type, an anti-EGFR therapy. | 27/09/2012 | FDA | Phase III RCT - Placebo Controlled | OS | 4 |
|  | For the treatment of locally advanced, unresectable or metastatic gastrointestinal stromal tumor (GIST) who have been previously treated with imatinib mesylate and sunitinib malate | 25/02/2013 | FDA | Phase III RCT - Placebo Controlled | PFS | 3 |
|  | For the treatment of hepatocellular carcinoma (HCC) who have been previously treated with sorafenib | 27/04/2017 | FDA | Phase III RCT - Placebo Controlled | OS | 4 |
| Ramucirumab | For the treatment of advanced gastric cancer or gastro-oesophageal junction adenocarcinoma after prior chemotherapy | 21/04/2014 | FDA | Phase III RCT - Placebo Controlled | OS | 4 |
|  | In combination with docetaxel, for treatment of metastatic non small cell lung cancer with disease progression on or after platinum-based chemotherapy. | 12/12/2014 | FDA | Phase III RCT - Placebo Controlled | OS | 4 |
|  | In combination with FOLFIRI, for the treatment of metastatic colorectal cancer with disease progression on or after prior therapy with bevacizumab, oxaliplatin, and a fluoropyrimidine. | 24/04/2015 | FDA | Phase III RCT - Placebo Controlled | OS | 4 |
| Avelumab | For the treatment of adult patients with metastatic Merkel cell carcinoma (MCC) | 23/03/2017 | FDA | Single Arm Phase II | BOR | 1 |
|  | For the treatment of patients with locally advanced or metastatic urothelial carcinoma (UC) who have disease progression during or following platinum-containing chemotherapy etastatic urothelial carcinoma (UC) | 09/05/2017 | FDA | Phase 1 Trial | ORR | 1 |
| Atezolizumab | For the treatment of patients with locally advanced or metastatic urothelial carcinoma who have disease progression during or following platinum-containing chemotherapy. | 18/05/2016 | FDA | Phase III RCT - Active Comparator | ORR | 1 |
|  | For the treatment of metastatic non-small cell lung cancer who have disease progression during or following platinum-containing chemotherapy. | 18/10/2016 | FDA | Phase III RCT - Active Comparator | OS | 5 |
|  | In combination with bevacizumab, paclitaxel, and carboplatin, for the first line treatment, of patients with metastatic non-squamous NSCLC with no  EGFR or ALK genomic tumor aberrations | 06/12/2018 | FDA | Phase III RCT - Active Comparator | OS, PFS | 5 |
| Eribulin | For the treatment of patients with metastatic breast cancer who have previously received at least two chemotherapeutic regimens for the treatment of metastatic disease. | 15/11/2010 | FDA | Phase III RCT - Active Comparator | OS | 4 |
|  | For the treatment of unresectable or metastatic liposarcoma who have received a prior anthracycline-containing regimen. | 28/01/2016 | FDA | Phase III RCT - Active Comparator | OS | 4 |
| Ruxolitinib | for treatment of patients with intermediate or high-risk myelofibrosis, including primary myelofibrosis, post-polycythemia vera myelofibrosis and post-essential thrombocythemia myelofibrosis. | 16/11/2011 | FDA | Phase III RCT - Placebo Controlled | Spleen volume reduction | 1 |
|  | For the treatment of polycythemia vera who have had an inadequate response to or are intolerant of hydroxyurea | 04/12/2014 | FDA | Phase III RCT - Active Comparator | PR | 1 |
| Nivolumab | For the treatment of unresectable or metastatic melanoma and disease progression following ipilimumab and, if BRAF V600 mutation positive, a BRAF inhibitor | 22/12/2014 | FDA | Phase III RCT - Active Comparator | OS | 4 |
|  | For the treatment of metastatic squamous non-small cell lung cancer with progression on or after platinum-based chemotherapy | 04/03/2015 | FDA | Phase III RCT - Active Comparator | OS | 5 |
|  | For the treatment of metastatic non-small cell lung cancer in patients with progression on or after platinum-based chemotherapy | 09/10/2015 | FDA | Phase 1 Trial | OS | 4 |
|  | For the treatment of advanced renal cell carcinoma in patients who have received prior antiangiogenic therapy | 23/11/2015 | FDA | Phase III RCT - Active Comparator | OS | 5 |
|  | In combination with ipilimumab, for the treatment of patients with BRAF V600 wild-type unresectable or metastatic melanoma | 23/11/2015 | FDA | Phase III RCT - Active Comparator | PFS | 3 |
|  | For the treatment of classical hodgkin lymphoma that has relapsed or progressed after autologous hematopoietic stem cell transplantation (HSCT) and post transplantation brentuximab vedotin | 17/05/2016 | FDA | Single Arm Phase II | ORR | 1 |
|  | For the treatment of recurrent or metastatic squamous cell carcinoma of the head and neck with disease progression on or after a platinum-based therapy | 10/11/2016 | FDA | Phase III RCT - Active Comparator | OS | 5 |
|  | For the treatment of locally advanced or metastatic urothelial carcinoma who have disease progression during or following platinum-containing chemotherapy or have disease progression within 12 months of neoadjuvant or adjuvant treatment with platinum-containing chemotherapy | 02/02/2017 | FDA | Single Arm Phase II | ORR | 1 |
|  | For the treatment of adult and pediatric (12 years and older) patients with microsatellite instability-high (MSI-H) or mismatch repair deficient (dMMR) metastatic colorectal cancer that has progressed following treatment with a fluoropyrimidine, oxaliplatin, and irinotecan, as a single agent or in combination with ipilimumab. | 31/07/2017 | FDA | Single Arm Phase II | ORR | 1 |
|  | For the treatment of hepatocellular carcinoma who have been previously treated with sorafenib | 22/09/2017 | FDA | Single Arm Phase II | ORR | 1 |
|  | For the treatment of melanoma with lymph node involvement or metastatic disease who have undergone complete resection | 20/12/2017 | FDA | Phase III RCT - Active Comparator | RFS | 1 |
|  | For the treatment of intermediate or poor risk, previously untreated advanced renal cell carcinoma, in combination with ipilimumab | 16/04/2018 | FDA | Phase III RCT - Active Comparator | OS, PFS, ORR | 5 |
|  | For the treatment of patients with metastatic small cell lung cancer with progression after platinum-based chemotherapy and at least one other line of therapy | 16/08/2018 | FDA | Single Arm Phase II | ORR | 1 |
| Pembrolizumab | For the treatment of patients with unresectable or metastatic melanoma and disease progression following ipilimumab and, if BRAF V600 mutation positive, a BRAF inhibitor | 04/09/2014 | FDA | Phase III RCT - Active Comparator | PFS, OS | 4 |
|  | For the treatment of metastatic NSCLC whose tumors express PD-L1 and who have disease progression on or after platinum-containing chemotherapy | 02/10/2015 | FDA | Phase III RCT - Active Comparator | PFS, OS | 5 |
|  | For the treatment of recurrent or metastatic HNSCC with disease progression on or after platinum-containing chemotherapy | 05/08/2016 | FDA | Phase III RCT - Active Comparator | OS | 4 |
|  | For previously untreated patients with locally advanced or metastatic Non-Small Cell Lung Cancer (NSCLC) whose tumors express PD-L1 | 24/10/2016 | FDA | Phase III RCT - Active Comparator | PFS | 3 |
|  | For the treatment of adult and pediatric patients with refractory cHL, or who have relapsed after 3 or more prior lines of therapy | 14/03/2017 | FDA | Single Arm Phase II | ORR | 1 |
|  | In combination with pemetrexed and carboplatin, for the first-line treatment of patients with metastatic non-squamous NSCLC | 10/05/2017 | FDA | Phase III RCT - Placebo Controlled | OS, PFS | 5 |
|  | For the treatment of 2nd line Metastatic Urothelial Carcinoma | 18/05/2017 | FDA | Phase III RCT - Active Comparator | OS, PFS | 4 |
|  | For the treatment of locally advanced or metastatic urothelial carcinoma who are not eligible for cisplatin-containing chemotherapy | 18/05/2017 | FDA | Single Arm Phase II | ORR | 1 |
|  | For the treatment of adult and pediatric patients with unresectable or metastatic, microsatellite instability-high (MSI-H) or mismatch repair deficient solid tumors that have progressed following prior treatment and who have no satisfactory alternative treatment options, or colorectal cancer that has progressed following treatment with a fluoropyrimidine, oxaliplatin, and irinotecan | 23/05/2017 | FDA | Single Arm Phase II | ORR | 1 |
|  | For the treatment of patients with recurrent locally advanced or metastatic gastric or gastroesophageal junction adenocarcinoma whose tumors express PD-L1 | 22/09/2017 | FDA | Controlled Phase II | ORR | 5 |
|  | For the treatment of patients with recurrent or metastatic cervical cancer with disease progression on or after chemotherapy whose tumors express PD-L1 | 12/06/2018 | FDA | Single Arm Phase II | ORR | 1 |
|  | For the treatment of refractory PMBCL, or for patients who have relapsed after 2 or more prior lines of therapy | 13/06/2018 | FDA | Single Arm Phase II | ORR | 1 |
|  | Adjuvant treatment of melanoma with involvement of lymph node(s) following complete resection | 18/10/2018 | EMA | Phase III RCT - Placebo Controlled | RFS | A |
|  | In combination with carboplatin and either paclitaxel or nabpaclitaxel, as first-line treatment of patients with metastatic squamous NSCLC. | 30/10/2018 | FDA | Phase III RCT - Active Comparator | PFS, OS | 5 |
|  | For the treatment of patients with HCC who have been previously treated with sorafenib | 09/11/2018 | FDA | Single Arm Phase II | ORR | 1 |
|  | For the treatment of adult and pediatric patients with recurrent locally advanced or metastatic Merkel cell carcinoma | 19/12/2018 | FDA | Single Arm Phase II | ORR | 1 |
| Brentuximab vedotin | For the treatment of patients with Hodgkin lymphoma after failure of autologous stem cell transplant (ASCT) or after failure of at least two prior multi-agent chemotherapy regimens in patients who are not ASCT candidates | 19/08/2011 | FDA | Single Arm Phase II | ORR | 1 |
|  | The treatment of patients with systemic anaplastic large cell lymphoma after failure of at least one prior multi-agent chemotherapy regimen | 19/08/2011 | FDA | Single Arm Phase II | ORR | 1 |
|  | For the treatment of classical HL at high risk of relapse or progression as post-auto-HSCT consolidation. | 17/08/2015 | FDA | Phase III RCT - Placebo Controlled | PFS | 3 |
|  | For the treatment of primary cutaneous anaplastic large cell lymphoma (pcALCL) or CD30­ expressing mycosis fungoides (MF) who have received prior systemic therapy | 09/11/2017 | FDA | Phase III RCT - Active Comparator | ORR | 1 |
|  | Previously untreated Stage III or IV classical Hodgkin lymphoma (cHL), in combination with chemotherapy | 20/03/2018 | FDA | Phase III RCT - Active Comparator | ORR | 1 |
|  | Previously untreated systemic anaplastic large cell lymphoma (sALCL) or other CD30-expressing peripheral T-cell lymphomas (PTCL), including angioimmunoblastic T-cell lymphoma and PTCL not otherwise specified, in combination with cyclophosphamide, doxorubicin, and prednisone | 16/11/2018 | FDA | Phase III RCT - Active Comparator | PFS | 3 |
| Ipilimumab | For the treatment of unresectable or metastatic melanoma | 25/03/2011 | FDA | Phase III RCT - Active Comparator | OS | 4 |
|  | Adjuvant treatment of patients with cutaneous melanoma with pathologic  involvement of regional lymph nodes of more than 1 mm who have  undergone complete resection, including total lymphadenectomy | 28/10/2015 | FDA | Phase III RCT - Placebo Controlled | RFS | 1 |
|  | In combination with nivolumab, for the treatment of patients with BRAF V600 wild-type unresectable or metastatic melanoma | 23/11/2015 | FDA | Phase III RCT - Active Comparator | PFS | 3 |
|  | For the treatment of patients with intermediate or poor risk, previously untreated advanced renal cell carcinoma, in combination with nivolumab | 16/04/2018 | FDA | Phase III RCT - Active Comparator | ORR,OS,PFS | 4 |
|  | For the treatment of adult and pediatric patients 12 years of age and older with microsatellite instability-high (MSI-H) or mismatch repair deficient (dMMR) metastatic colorectal cancer that has progressed following treatment with a fluoropyrimidine, oxaliplatin, and irinotecan, in combination with nivolumab | 10/07/2018 | FDA | Controlled Phase II | ORR | 1 |
| Romidepsin | Treatment of cutaneous T-cell lymphoma (CTCL) in patients who have received at least one prior systemic therapy | 1/05/2009 | FDA | Single Arm Phase II | ORR | 1 |
|  | Treatment of peripheral T-cell lymphoma (PTCL) in patients who have received at least one prior therapy | 16/06/2011 | FDA | Single Arm Phase II | ORR | 1 |
| Vemurafenib | For the treatment of patients with unresectable or metastatic melanoma with BRAFV600E mutation | 17/08/2011 | FDA | Phase III RCT - Active Comparator | OS, PFS | 4 |
|  | For the treatment of patients with ErdheimChester Disease with BRAF V600 mutation. | 06/11/2017 | FDA | Single Arm Phase II | ORR | 1 |
| Lenvatinib | For the treatment of patients with locally recurrent or metastatic, progressive, radioactive iodine-refractory differentiated thyroid cancer | 13/02/2015 | FDA | Phase III RCT - Placebo Controlled | PFS | 2 |
|  | For the treatment of Renal Cell Cancer (RCC): in combination with everolimus, for patients with advanced RCC following one prior anti-angiogenic therapy. | 13/05/2016 | FDA | Controlled Phase II | PFS | 2 |
|  | For the first-line treatment of patients with unresectable hepatocellular carcinoma (HCC). | 15/08/2018 | FDA | Phase III RCT - Active Comparator | OS | 2 |
| **MEDICINES WITH MULTIPLE INDICATIONS ACROSS DIFFERENT LINES OF THERAPY** | | | | | | |
| Abiraterone Acetate | For the treatment of patients with metastatic castration-resistant  prostate cancer who have received prior chemotherapy containing docetaxel | 28/04/2011 | FDA | Phase III RCT - Placebo Controlled | OS | 5 |
|  | For the treatment of metastatic castration resistant prostate cancer in adult men who are asymptomatic or mildly symptomatic after failure of androgen deprivation therapy. | 15/11/2012 | EMA | Phase III RCT - Placebo Controlled | PFS, OS | 5 |
|  | For the treatment of newly diagnosed high risk metastatic hormone sensitive prostate cancer (mHSCP) in adult men in combination with androgen deprivation therapy (ADT) for Zytiga plus prednisone or pednisolone | 12/10/2017 | EMA | Phase III RCT - Active Comparator | PFS, OS | 5 |
| Afatinib | For the first-line treatment of patients with metastatic non-small cell lung cancer (NSCLC) whose tumors have epidermal growth factor receptor (EGFR) exon 19 deletions or exon 21 (L858R) substitution mutations | 12/07/2013 | FDA | Phase III RCT - Active Comparator | PFS | 3 |
|  | Treatment of patients with locally advanced or metastatic NSCLC of squamous histology progressing on or after platinum-based chemotherapy | 25/02/2016 | EMA | Phase III RCT - Active Comparator | PFS | 2 |
| Blinatumomab | For the treatment of philadelphia chromosome-negative relapsed or refractory B-cell precursor acute lymphoblastic leukaemia (ALL). | 03/12/2014 | FDA | Single Arm Phase II | Rate of CR | 1 |
|  | For the treatment of relapsed or refractory B-cell precursor acute lymphoblastic leukemia (ALL) in adults and children | 11/07/2017 | FDA | Single Arm Phase II | Rate of CR | 1 |
|  | For the treatment of B-cell precursor acute lymphoblastic leukemia (ALL) in first or second complete remission | 05/03/2018 | FDA | Single Arm Phase II | MRD rate | 1 |
| Enzalutamide | For the treatment of patients with metastatic castration-resistant prostate cancer who have previously received docetaxel. | 31/08/2012 | FDA | Phase III RCT - Placebo Controlled | OS | 5 |
|  | For the treatment of patients with metastatic castration-resistant prostate cancer. | 10/09/2014 | FDA | Phase III RCT - Placebo Controlled | OS, PFS | 4 |
|  | For the treatment of patients with castration-resistant prostate cancer | 13/07/2018 | FDA | Phase III RCT - Placebo Controlled | MFS | 1 |
| Rucaparib | For the treatment of patients with deleterious BRCA mutation  (germline and/or somatic) associated advanced ovarian cancer who have been  treated with two or more chemotherapies | 19/12/2016 | FDA | Single Arm Phase II | ORR | 1 |
|  | For the treatment of recurrent epithelial ovarian, fallopian tube, or primary peritoneal cancer who are in a complete or partial response to platinum-based chemotherapy | 06/04/2018 | FDA | Phase III RCT - Placebo Controlled | PFS | 3 |
| Osimertinib | For the treatment of patients with metastatic epidermal growth factor receptor (EGFR) T790M mutationpositive non-small cell lung cancer (NSCLC) | 13/11/2015 | FDA | Single Arm Phase II | ORR | 1 |
|  | First-line treatment of patients with metastatic NSCLC whose tumors  have epidermal growth factor receptor (EGFR) exon 19 deletions or exon  21 L858R mutations | 18/04/2018 | FDA | Phase III RCT - Active Comparator | PFS | 3 |
| Crizotinib | For the treatment of previously treated anaplastic lymphoma kinase (ALK)-positive advanced non-small cell lung cancer (NSCLC) | 26/08/2011 | FDA | Phase 1 Trial | ORR | 1 |
|  | First-line treatment of adults with anaplastic lymphoma kinase (ALK)-positive advanced non-small cell lung cancer (NSCLC) | 27/09/2013 | TGA | Phase III RCT - Active Comparator | PFS | 3 |
|  | For the treatment of metastatic NSCLC whose tumors are ROS1-positive | 11/03/2016 | FDA | Phase 1 Trial | ORR | 1 |
| Bosutinib | For the treatment of adult patients with chronic, accelerated, or blast phase Ph+ chronic myelogenous leukemia (CML) with resistance or intolerance to prior therapy | 04/09/2012 | FDA | Single Arm Phase II | MCyR | 1 |
|  | For the treatment of newly-diagnosed chronic phase Ph+ chronic myelogenous leukemia (CML) | 19/12/2017 | FDA | Phase III RCT - Active Comparator | MMR | 1 |
| Alectinib | For the treatment of patients with anaplastic lymphoma kinase (ALK)-positive, metastatic non-small cell lung cancer (NSCLC) who have progressed on or are intolerant to crizotinib | 11/12/2015 | FDA | Single Arm Phase II | ORR | 1 |
|  | For the treatment of anaplastic lymphoma kinase (ALK)-positive metastatic non-small cell lung cancer (NSCLC) | 12/10/2017 | EMA | Phase III RCT - Active Comparator | PFS | 3 |
| Ceritinib | For the treatment of patients with anaplastic lymphoma kinase (ALK)-positive metastatic non-small cell lung cancer (NSCLC) who have progressed on or are intolerant to crizotinib. | 29/04/2014 | FDA | Phase 1 Trial | ORR, DOR | 1 |
|  | First-line treatment of adult patients with anaplastic lymphoma kinase (ALK)-positive advanced non-small cell lung cancer (NSCLC) | 18/05/2017 | EMA | Phase III RCT - Active Comparator | PFS | 3 |
| Ofatumumab | For the treatment of patients with chronic lymphocytic leukemia (CLL) refractory to fludarabine and alemtuzumab | 26/10/2009 | FDA | Single Arm Phase II | ORR | 1 |
|  | in combination with chlorambucil, for the treatment of previously untreated patients with chronic lymphocytic leukemia (CLL) for whom fludarabine-based therapy is considered inappropriate | 17/04/2014 | FDA | Phase III RCT - Active Comparator | PFS | 3 |
|  | for extended treatment of patients who are in complete or partial response after at least two lines of therapy for recurrent or progressive CLL. | 19/01/2016 | FDA | Phase III RCT - Placebo Controlled | PFS | 3 |
|  | in combination with fludarabine and cyclophosphamide for the treatment of patients with relapsed CLL | 30/08/2016 | FDA | Phase III RCT - Active Comparator | PFS | 3 |

Abbreviations: BCVA - best-corrected visual acuity; BOR - best observed response; CR – complete response rate; DOR - duration of response; FVC - forced vital capcity; MCyR - major cytogenic response; MMR - major molecular response; ORR - overall response rate; OS – overall survival; PFS - progression free survival; PR – primary response; RFS -remission free survival,, SEGA - subependymal giant cell astrocytoma

**TABLE C2 – ALIGNMENT OF REGULATORY APPROVAL AND HTA APPROVAL SEQUENCE**

| **Molecule** | **Approved Indications (Brief version)** | **Regulatory Approval Sequence** | **HTA Approval Sequence** | | | | | |
| --- | --- | --- | --- | --- | --- | --- | --- | --- |
|  |  |  | **NICE** | **SMC** | **HAS** | **G-BA** | **CADTH** | **PBAC** |
| **Ibrutinib** | 2nd line Mantle Cell Lymphoma  2^nd^ Line Chronic lymphocytic leukemia  1^st^ Line Chronic lymphocytic leukemia  Waldenström’s macroglobulinemia  2nd Line Marginal zone lymphoma  Chronic graft versus host disease | **1**  **2**  **3**  **4**  **5**  **6** | 3^rd^  1^st^  NS  2^nd^  NM  NM | 1^st^  2^nd^  NS  3^rd^  NM  NM | 1^st^  2^nd^  3^rd^  4^th^  NM  NM | 1^st^  2^nd^  3^rd^  NAB  NM  NM | 2^nd^  1^st^  3^rd^  DNL  NS  NS | 2^nd^  1^st^  3^rd^  NS  NM  NM |
| **Nintedanib** | 2nd line NSCLC  Idiopathic Pulmonary Fibrosis | **1**  **2** | 1^st^  2^nd^ | 1^st^  2^nd^ | INS  1^st^ | 1^st^  2^nd^ | NM  1^st^ | DNL  1^st^ |
| **Aflibercept** | Neovascular Age-Related Macular Degeneration  Combination metastatic colorectal cancer  Central Retinal Vein Occlusion  Diabetic Macular Edema  Branch Retinal Vein Occlusion  Diabetic Retinopathy  Myopic choroidal neovascularisation | **1**  **2**  **3**  **4**  **5**  **6**  **7** | 1^st^  DNL  2^nd^  3^rd^  5^th^  NM  4^th^ | 1^st^  2^nd^  3^rd^  4^th^  5^th^  NM  6^th^ | 1^st^  2^nd^  3^rd^  4^th^  5^th^  NM  6^th^ | NAB  1^st^  NAB  NAB  NAB  NM  NAB | 1^st^  DNL  T 2^nd^  T 2^nd^  4^th^  NM  NS | 1^st^  DNL  2^nd^  3^rd^  4^th^  NM  5^th^ |
| **Everolimus** | Advanced renal cell carcinoma  Subependymal giant cell astrocytoma  Pancreatic progressive neuroendocrine tumors  Renal angiomyolipoma and tuberous sclerosis complex  Advanced breast cancer  Neuroendocrine tumors of GI or lung origin  Tuberous sclerosis complex associated seizures | **1**  **2**  **3**  **4**  **5**  **6**  **7** | 2^nd^  NS  T 3^rd^  NS  1^st^  T 3^rd^  NS | DNL  NS  1^st^  NS  2^nd^  3^rd^  NS | 1^st^  2^nd^  3^rd^  4^th^  5^th^  6^th^  7^th^ | 1^st^  NS  NS  NS  NS  NS  NS | NS  DNL  1^st^  NS  2^nd^  3^rd^  NS | T 3^rd^  1^st^  T 3^rd^  NM  2^nd^  NS  5^th^ |
| **Cabozantinib** | Metastatic medullary thyroid cancer  2^nd^ line renal cell carcinoma  1^st^ line renal cell carcinoma  2^nd^ line hepatocellular carcinoma | **1**  **2**  **3**  **4** | 2^nd^  1^st^  3^rd^  NS | DNL  1^st^  DNL  NS | 1^st^  2^nd^  INS  3^rd^ | 1^st^  2^nd^  NAB  3^rd^ | NM  1^st^  NS  2^nd^ | NM  1^st^  DNL  DNL |
| **Pazopanib** | Advanced renal cell carcinoma  Advanced soft tissue sarcoma | **1**  **2** | 1^st^  NS | 1^st^  DNL | INS  1^st^ | NS  NS | 1^st^  DNL | 1^st^  2^nd^ |
| **Tisagenlecleucel*** | R/R acute lymphoblastic leukemia  R/R diffuse large B-cell lymphoma | **1**  **2** | 1^st^  2^nd^ | 1^st^  2^nd^ | 1^st^  2^nd^ | 1^st^  2^nd^ | NS  NS | NS  NS |
| **Regorafenib** | 2nd Line metastatic colorectal cancer  2nd Line metastatic gastrointestinal stromal tumor  2nd Line hepatocellular carcinoma | **1**  **2**  **3** | NS  1^st^  2^nd^ | NS  1^st^  2^nd^ | 1^st^  2^nd^  3^rd^ | 1^st^  NAB  2^nd^ | DNL  1^st^  2^nd^ | DNL  DNL  DNL |
| **Ramucirumab** | 2^nd^ line gastric cancer  2nd Line NSCLC  2nd Line metastatic colorectal cancer | **1**  **2**  **3** | DNL  DNL  NS | NS  NS  NS | 1^st^  NS  2^nd^ | 1^st^  NAB  NAB | 1^st^  NM  NM | 1^st^  NM  NM |
| **Avelumab** | Metastatic Merkel cell carcinoma  Metastatic urothelial carcinoma | **1**  **2** | 1^st^  NM | 1^st^  NM | 1^st^  NM | 1^st^  NM | 1^st^  NS | 1^st^  NM |
| **Atezolizumab** | 2nd Line urothelial carcinoma  2nd Line NSLCLC  Combination 1st Line NSCLC | **1**  **2**  **3** | 1^st^  2^nd^  3^rd^ | DNL  1^st^  DNL | NS  1^st^  2^nd^ | 2^nd^  1^st^  NAB | NS  1^st^  DNL | NS  1^st^  2^nd^ |
| **Eribulin** | 3^rd^ line metastatic breast cancer  2^nd^ line liposarcoma | **1**  **2** | 1^st^  NS | 1^st^  NS | 1^st^  2^nd^ | 1^st^  NAB | 1^st^  NS | 1^st^  2^nd^ |
| **Ruxolitinib** | Myelofibrosis  Polycythemia vera | **1**  **2** | 1^st^  NS | 1^st^  NS | 1^st^  2^nd^ | 1^st^  2^nd^ | 1^st^  2^nd^ | 1^st^  NS |
| **Nivolumab** | 2nd Line Melanoma  2^nd^ Line squamous NSCLC  2^nd^ Line non-squamous NSCLC  2nd Line Renal Cell Carcinoma  Combination 1st Line Melanoma  Classical Hodgkin Lymphoma  Metastatic squamous cell carcinoma of the head and neck  2nd Line Urothelial carcinoma  Combination Microsatellite Instability-High Cancer  Hepatocellular carcinoma  Adjuvant Melanoma  Combination 1st Line Renal Cell Carcinoma  2nd Line SCLC | **1**  **2**  **3**  **4**  **5**  **6**  **7**  **8**  **9**  **10**  **11**  **12**  **13** | 1^st^  T 5th  T 5th  3^rd^  2^nd^  4^th^  7^th^  DNL  NM  NM  8^th^  9^th^  NM | 2^nd^  1^st^  3^rd^  5^th^  4^th^  6^th^  7^th^  DNL  NM  NM  8^th^  9^th^  NM | 1^st^  T 3^rd^  T 3^rd^  2^nd^  6^th^  5^th^  7^th^  NS  NM  NM  8^th^  9^th^  NM | 1^st^  2^nd^  T 3^rd^  T 3^rd^  NAB  NAB  5^th^  NAB  NM  NM  6^th^  7^th^  NM | 1^st^  2^nd^  NS  3^rd^  5^th^  6^th^  4^th^  NM  NM  DNL  7^th^  8^th^  NM | 1^st^  T 2^nd^  T 2^nd^  T 2^nd^  6^th^  NS  5^th^  NS  NM  NS  DNL  NS  NM |
| **Pembrolizumab** | 2nd Line Metastatic Melanoma  2nd Line Metastatic NSCLC  2nd Line Metastatic HNSCC  1st line Metastatic NSCLC  Relapsed/Refractory classical Hodgkin Lymphoma  Combination 1st Line Non-squamous NSCLC  2nd line Metastatic Urothelial Carcinoma  1st line Metastatic Urothelial Carcinoma  2nd Line - Microsatellite Instability-High Cancer  3rd line Metastatic Gastric Cancer  2nd line Cervical Cancer  3rd line Primary Mediastinal Large B-Cell Lymphoma  Adjuvant Treatment Melanoma  Combination 1st Line Squamous NSCLC  2nd Line Hepatocellular Carcinoma  Metastatic Merkell Cell Carcinoma | **1**  **2**  **3**  **4**  **5**  **6**  **7**  **8**  **9**  **10**  **11**  **12**  **13**  **14**  **15**  **16** | 1^st^  2^nd^  NS  3^rd^  6^th^  NS  4^th^  5^th^  NS  NM  NM  NM  7^th^  8^th^  NM  NM | 1^st^  2^nd^  9^th^  3^rd^  5^th^  NS  4^th^  DNL  NS  NM  NM  NM  7^th^  8^th^  NM  NM | 1^st^  2^nd^  6^th^  3^rd^  5^th^  NS  4^th^  NS  NS  NM  NM  NM  7^th^  8^th^  NM  NM | 1^st^  2^nd^  NAB  3^rd^  NAB  T 5^th^  4^th^  NAB  NS  NM  NM  NM  T 5^th^  T 5^th^  NM  NM | 1^st^  2^nd^  NM  3^rd^  4^th^  6^th^  5^th^  DNL  NS  NM  NM  NM  7^th^  8^th^  NM  NM | 1^st^  DNL  DNL  T 3^rd^  2^nd^  DNL  T 3^rd^  NS  DNL  NM  NM  T 6^th^  T 6^th^  5^th^  NM  NM |
| **Brentuximab vedotin** | 3rd line Hodgkin lymphoma  2nd line systemic anaplastic large cell lymphoma  Classical Hodgkin lymphoma at high risk of relapse  Primary cutaneous anaplastic large cell lymphoma  1st line classical Hodgkin lymphoma  1st line systemic anaplastic large cell lymphoma | **1**  **1**  **3**  **4**  **5**  **6** | 1^st^  2^nd^  NS  3^rd^  NS  NM | T 1^st^  T 1^st^  NS  3^rd^  NS  NM | T 1^st^  T 1^st^  3^rd^  4^th^  INS  NM | T 1^st^  T 1^st^  NAB  3^rd^  NAB  NM | 1^st^  2^nd^  3^rd^  T 5^th^  T 5^th^  4^th^ | T 2^nd^  1^st^  T 2^nd^  4^th^  NM  NM |
| **Ipilimumab** | Advanced melanoma  Adjuvant treatment of melanoma  Combination melanoma  Combination renal cell carcinoma  Combination Microsatellite Instability-High Cancer | **1**  **2**  **3**  **4**  **5** | 1^st^  NM  2^nd^  3^rd^  NM | 1^st^  NM  2^nd^  3^rd^  NM | 1^st^  NM  2^nd^  3^rd^  NM | 1^st^  NM  NAB  3^rd^  NM | 1^st^  NM  2^nd^  3^rd^  NM | 1^st^  NM  2^nd^  3^rd^  NM |
| **Romidepsin** | Cutaneous T-cell lymphoma  Peripheral T-cell lymphoma | **1**  **2** | NM  NM | NM  NM | NM  NM | NM  NM | NM  1^st^ | NM  DNL |
| **Vemurafenib** | Metastatic melanoma  Erdheim-Chester Disease | **1**  **2** | 1^st^  NM | 1^st^  NM | 1^st^  NM | 1^st^  NM | NS  NM | DNL  NM |
| **Lenvatanib** | Thyroid cancer  Renal Cell Carcinoma  Hepatocellular carcinoma | **1**  **2**  **3** | 2^nd^  1^st^  3^rd^ | 1^st^  3^rd^  2^nd^ | 1^st^  INS  INS | 1^st^  2^nd^  NAB | 1^st^  DNL  2^nd^ | 1^st^  DNL  2^nd^ |
| **Abiraterone Acetate** | 2nd line metastatic castrate-resistant prostate cancer  1st line metastatic castrate resistant prostate cancer  Metastatic high-risk castration-sensitive prostate cancer | **1**  **2**  **3** | 1^st^  2^nd^  NS | 1^st^  2^nd^  3^rd^ | 1^st^  2^nd^  3^rd^ | 1^st^  2^nd^  3^rd^ | NS  1^st^  NS | 1^st^  DNL  NS |
| **Afatinib** | 1^st^ line NSCLC  2^nd^ line NSCLC | **1**  **2** | 1^st^  NS | 1^st^  NS | 1^st^  INS | 1^st^  NAB | 1st  NS | 1^st^  DNL |
| **Blinatumomab** | R/R B-cell precursor ALL (Ph-)  R/R B-cell precursor ALL  First or second complete remission B-cell ALL | **1**  **2**  **3** | 1^st^  NS  2^nd^ | 1^st^  2^nd^  3^rd^ | 1^st^  2^nd^  NS | 1^st^  2^nd^  3^rd^ | 1^st^  NS  3^rd^ | 1^st^  NS  3^rd^ |
| **Enzalutamide** | 2nd line metastatic castrate-resistant prostate cancer  1st line metastatic castrate resistant prostate cancer  Castrate resistant prostate cancer | **1**  **2**  **3** | 1^st^  2^nd^  DNL | 1^st^  2^nd^  DNL | 1^st^  2^nd^  3^rd^ | 1^st^  2^nd^  NAB | 1^st^  2^nd^  3^rd^ | 1^st^  DNL  NS |
| **Rucaparib** | 3rd Line Ovarian, Fallopian, Peritoneal Cancer  Maintenance treatment ovarian, fallopian, peritoneal Cancer | **1**  **2** | NS  1^st^ | NS  1^st^ | NS  1^st^ | NAB  NAB | NM  NM | NM  NM |
| **Osimertinib** | 1^st^ line NSCLC  2^nd^ line NSCLC | **1**  **2** | 1^st^  DNL | 1^st^  DNL | 1^st^  2^nd^ | NAB  1^st^ | 1^st^  2^nd^ | 1^st^  DNL |
| **Crizotinib** | 2^nd^ line NSCLC  1^st^ line NSCLC  1^st^ line NSCLC (ROS1 +) | **1**  **2**  **3** | 2^nd^  1^st^  3^rd^ | 1^st^  2^nd^  3^rd^ | 1^st^  2^nd^  3^rd^ | 1^st^  2^nd^  NAB | 1^st^  NM  2^nd^ | 1^st^  NS  2^nd^ |
| **Bosutinib** | 2nd Line Ph+ chronic myelogenous leukemia  1st Line Ph+ chronic myelogenous leukemia | **1**  **2** | 1^st^  NS | 1^st^  NS | 1^st^  2^nd^ | 1^st^  NAB | 1^st^  NS | 1^st^  NM |
| **Alectinib** | 2^nd^ line NSCLC  1^st^ line NSCLC | **1**  **2** | NS  1^st^ | NS  1^st^ | 1^st^  2^nd^ | 1^st^  2^nd^ | 1^st^  2^nd^ | 1^st^  NS |
| **Ceritinib** | 2^nd^ line NSCLC  1^st^ line NSCLC | **1**  **2** | 1^st^  2^nd^ | 1^st^  NS | 1^st^  2^nd^ | NAB  NAB | 1^st^  NS | 1^st^  NS |
| **Ofatumumab** | 2^nd^ line chronic lymphocytic leukemia  Combination 1^st^ line chronic lymphocytic leukemia  Maintenance treatment chronic lymphocytic leukemia  Combination 2^nd^ line chronic lymphocytic leukemia | **1**  **2**  **3**  **4** | NM  NM  NM  NM | NM  NM  NM  NM | NM  NM  NM  NM | NM  NM  NM  NM | NM  NM  NM  NM | NS  1^st^  NM  NM |

Abbreviations: ALL – acute lymphoblastic leukemia; NSCLC – Non-small cell lung cancer.. HNSCS – Head and Neck Squamous cell carcinoma.

Legend: NS = No submission (Indicates that no HTA decision was identified for this indication during the study period); NM = Not marketed (indication does not have marketing authorisation within respective jurisdiction); DNL = Do not list (HTA agency in England, Scotland, Canada or Australia issued a negative coverage recommendation), INS = Insufficient (The HAS in France gave an SMR rating of insufficient), NAB = No added Benefit (the G-BA in Germany gave a rating of no proof of added benefit).
